# The first complete mitochondrial genome of the Indian Tent Turtle, *Pangshura tentoria* (Testudines: Geoemydidae): Characterization and comparative analysis

**DOI:** 10.1002/ece3.5606

**Published:** 2019-08-30

**Authors:** Shantanu Kundu, Vikas Kumar, Kaomud Tyagi, Rajasree Chakraborty, Kailash Chandra

**Affiliations:** ^1^ Centre for DNA Taxonomy Molecular Systematics Division Zoological Survey of India Kolkata India

**Keywords:** evolution, freshwater turtles, genomics, mitogenome, *Pangshura*, phylogeny

## Abstract

The characterization of a complete mitogenome is widely used in genomics studies for systematics and evolutionary research. However, the sequences and structural motifs contained within the mitogenome of Testudines taxa have rarely been examined. The present study decodes the first complete mitochondrial genome of the Indian Tent Turtle, *Pangshura tentoria* (16,657 bp) by using next‐generation sequencing. This denovo assembly encodes 37 genes: 13 protein‐coding genes (PCGs), 22 transfer RNA (tRNAs), two ribosomal RNA, and one control region (CR). Most of the genes were encoded on majority strand, except for one PCG (NADH dehydrogenase subunit 6) and eight tRNAs. Most of the PCGs were started with an ATG initiation codon, except for Cytochrome oxidase subunit 1 with “GTG” and NADH dehydrogenase subunit 5 with “ATA.” The termination codons, “TAA” and “AGA” were observed in two subunits of NADH dehydrogenase gene. The relative synonymous codon usage analysis revealed the maximum abundance of alanine, isoleucine, leucine, and threonine. The nonsynonymous/synonymous ratios were <1 in all PCGs, which indicates strong negative selection among all Geoemydid species. The study also found the typical cloverleaf secondary structure in most of the tRNA genes, except for serine with the lack of the conventional DHU arm. The comparative study of Geoemydid mitogenomes revealed the occurrence of tandem repeats was frequent in the 3′ end of CR. Further, two copies of a unique tandem repeat “TTCTCTTT” were identified in *P. tentoria*. The Bayesian and maximum‐likelihood phylogenetic trees using concatenation of 13 PCGs revealed the close relationships of *P. tentoria* with *Batagur trivittata* in the studied dataset. All the Geoemydid species showed distinct clustering with high bootstrap support congruent with previous evolutionary hypotheses. We suggest that the generations of more mitogenomes of Geoemydid species are required, to improve our understanding of their in‐depth phylogenetic and evolutionary relationships.

## Graphical abstract

The present study decodes the first complete mitochondrial genome of the Indian Tent Turtle, *Pangshura tentoria* by using next‐generation sequencing. This denovo assembly encodes 37 genes: 13 protein coding genes (PCGs), 22 transfer RNA (tRNAs), two ribosomal RNA (rRNAs), and one control region (CR). The maximum‐likelihood (ML) phylogenetic trees using concatenation of 13 PCGs revealed the close relationships of *P. tentoria* with *Batagur trivittata*.

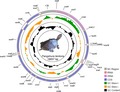

## 1 INTRODUCTION

Geoemydid turtles are an ornamental and highly threatened living group among Testudines in the world (Fritz & Havaš, 2007). This group is known by 71 extant species and is recognized as the sister taxa of land tortoises (Family Testudinidae; TTWG, 2017). Most Geoemydids are adapted to freshwater ecosystems; however, a few prefer estuarine and terrestrial habitats (Ernst, Altenburg, & Barbour, 2000). India is regarded as one of the turtle hotspots in the globe harboring 16 Geoemydid species (Buhlmann et al., 2009; Kundu, Kumar, Laskar, Tyagi, & Chandra, 2018). These species are distributed from the north to east, and up to the northeastern region of India, except *Vijayachelys silvatica*, which is a southern endemic (Deepak, Praschag, & Vasudevan, 2014). Among the Indian Geoemydids, *Pangshura* is one of the highly threatened genera with four extant species, *P. tecta*, *P. tentoria*, *P. sylhetensis*, *P. smithii* and one extinct species, *P. tatrotia* (Das, 2001; Walter & Tyler, 2010). Combined analysis of the fossil record, morphology, and molecular data indicated that the distribution of *P. tatrotia* included the Siwalik Hills of Pakistan during the Pliocene epoch (2.59–3.59 million years ago; Walter & Tyler, 2010). Further, the fossil records excavated from the Siwalik Hills and Narmada valley deposits suggested the existence of *Pangshura* in India since the Pleistocene epoch (Baruah, Devi, & Sharma, 2016).

In Testudines systematics, the genus *Pangshura* with four living species was for more than a century placed with three large‐sized *Kachuga* species (Das, 1991, 1995; Ernst & Barbour, 1989), but was re‐established based on morphological and molecular studies (Praschag, Hundsdörfer, & Fritz, 2007). The Indian Roofed turtle (*P. tecta*) and the Brown Roofed turtle (*P. smithii*) are found in Bangladesh, India, Nepal, and Pakistan; the Indian Tent Turtle (*P. tentoria*) is found in Bangladesh, India, and Nepal; and the Assam Roofed turtle (*P. sylhetensis*) is endemic to Bangladesh and India (TTWG, 2017; Figure S1). *P. tecta* and *P. tentoria* are categorized as “Lower Risk/Least concern,” whereas *P. sylhetensis* is categorized as “Endangered” and *P. smithii* as “Near Threatened” following guidelines from the International Union for Conservation of Nature (IUCN) Red data list (IUCN, 2019). Nevertheless, the populations of *P. tecta* and *P. tentoria* have dramatically declined in the northeastern region and other parts of India due to several anthropogenic threats, like illegal poaching and habitat loss (Van Dijk, 2000). Hence, *P. tentoria* is also listed as “Appendix II” category in the Convention on International Trade in Endangered Species of Wild Fauna and Flora (CITES) and recommended to be listed in Indian Wildlife (Protection) Act, 1972 (Ahmed & Das, 2010).

Molecular data have been widely employed in Testudines systematics research and conservation genetics (Murphy et al., 2013; Spitzweg, Praschag, DiRuzzo, & Fritz, 2018). Mitochondrial genes, nuclear genes, and microsatellite marker have been used for identifying new species (Fritz et al., 2008; Ihlow et al., 2016), recognizing the genetic diversity and population structure (Fritz, Gemel, Kehlmaier, Vamberger, & Praschag, 2014), and estimating the phylogeny and evolutionary relationships of Testudines (Le, Raxworthy, McCord, & Mertz, 2006). Complete mitogenomes have also been examined to understand the evolution of Testudines and provide evidence to suggest a sister relationship between turtles and archosaurs among amniotes (Kumazawa & Nishida, 1999; Zardoya & Meyer, 1998). Further, the structural characteristics of protein‐coding genes (PCGs), transfer RNA genes (tRNAs), ribosomal RNA genes (rRNAs), and control regions (CRs) and their arrangements were evaluated to demonstrate how some genomic features can adjudicate phylogenetic relationships (Mindell et al., 1999; Parham, Feldman, & Boore, 2006; San Mauro, Gower, Zardoya, & Wilkinson, 2006). However, the availability of Testudines mitogenomes is limited in global databases. Currently, 31 mitogenomes of species comprising seven Geoemydidae genera (*Mauremys*, *Cuora*, *Heosemys*, *Sacalia*, *Notochelys*, *Cyclemys*, and *Batagur*) are available in GenBank. Among them, only four mitogenomes (KX817298, DQ659152, KF574821, and JX455823) of four species (*Batagur trivittata*, *Cuora mouhotii*, *Cuora trifasciata*, and *Cyclemys dentata*) are published (Feng, Yang, Zhang, & Zhao, 2017; Huang et al., 2015; Li, Zhang, Zhao, Shi, & Zhu, 2015; Zhang, Nie, Cao, & Zhan, 2008). However, no complete mitogenome of any species of *Pangshura* is available. Therefore, the present study aimed to generate the mitogenome of *Pangshura tentoria* and perform comparative analysis with other Geoemydid species for insights into their evolutionary relationships.

## 2 MATERIALS AND METHODS

### 2.1 Ethics statement

Prior permission was acquired from the wildlife authority, the Arunachal Pradesh Biodiversity Board (Letter No. SFRI/APBB/09‐2011‐1221‐1228 dated 22.07.2016) and Zoological Survey of India, Kolkata (Letter No. ZSI/MSD/CDT/2016‐17 dated 29.07.2016) for the fieldwork and sampling. No turtle specimens were sacrificed in the current study. The sampling and analytical methods were carried out in accordance with appropriate guidelines, and best ethical and experimental practice of the Zoological Survey of India.

### 2.2 Sample collection, and DNA extraction

The fieldwork was conducted in the northeastern region of India, and a *P. tentoria* sample was collected from Arunachal Pradesh state (latitude 27°30′N and longitude 95°59′E; Figure S2). The blood sample was collected aseptically from the limbs by using a micro‐syringe and subsequently stored in EDTA containing vial at 4°C. The specimen was released back in the same environment after collecting the biological sample. About 10 µl of blood sample was centrifuged at 700 × *g* for 5 min at 4°C in 1 ml buffer (0.32 M Sucrose, 1 mM EDTA, 10 mM TrisHCl) to remove nuclei and cell debris. The supernatant was collected in 1.5 ml eppendorf tubes and centrifuged at 12,000 × *g* for 10 min at 4°C to precipitate the mitochondria. The mitochondrial pellet was resuspended in 200 µl of buffer (50 mM TrisHCl, 25 mM of EDTA, 150 mM NaCl), with the addition of 20 µl of proteinase K (20 mg/ml) followed by incubation at 56°C for 1 hr. Lastly, the mitochondrial DNA was extracted by Qiagen DNeasy Blood & Tissue Kit (QIAGEN Inc.). The DNA quality was checked in 1% agarose gel electrophoresis, and the concentration of mitochondrial DNA was quantified by NANODROP 2000 spectrophotometer (Thermo Scientific).

### 2.3 Mitogenome sequencing, assembly, and annotation

Complete mitochondrial genome sequencing and denovo assembly was carried out at Genotypic Technology Pvt. Ltd. Bangalore, India (http://www.genotypic.co.in/). First, 200 ng of DNA was used in Illumina TruSeq Nano DNA HT library preparation kit for library assembly (Illumina, Inc). The purified A‐tailed fragments were ligated with the sequencing indexed adapters after the fragmentation of mitochondrial DNA by ultrasonication (Covaris M220, Covaris Inc.). Then, fragments of 450 bp were selected using sample purification beads and amplified by polymerase chain reaction (PCR) to enrich it. The amplified PCR library was analyzed using a Bioanalyzer 2200 (Agilent Technologies, Inc.) with high sensitivity DNA chips. After obtaining the required concentration (632.77 pg/µl) and mean peak size (466 bp) opted in NEXTflex Rapid DNA protocol (BIOO Scientific), total >4 million raw reads were generated through Illumina NextSeq 500 (150 × 2 chemistry; Illumina Inc). The raw reads were processed using the cutadapt tool (http://code.google.com/p/cutadapt/) for adapters and low‐quality base trimming with a cutoff of Phred quality scores of Q20. Total sequencing depth was >71,000×. The high‐quality reads were down sampled to 2 million reads using Seqtk (https://github.com/lh3/seqtk) and down sampled high‐quality reads were denovo assembled using SPAdes‐3.7.1 using default parameters (Bankevich et al., 2012). The generated sequence annotation was also checked in MITOS online server (http://mitos.bioinf.uni-leipzig.de). The DNA sequences of PCGs were initially translated into the putative amino acid sequences on the basis of the genetic code of vertebrate mitochondrial genome. The mitogenome (accession no. MH795989) was submitted to the GenBank database through the Sequin submission tool (Figure S3).

### 2.4 Genome visualization, characterization, and comparative analysis

The circular representation of the generated mitogenome of *P. tentoria* was mapped by CGView Server (http://stothard.afns.ualberta.ca/cgview_server/) with default parameters (Grant & Stothard, 2008). Based on a homology search in the Refseq database (https://www.ncbi.nlm.nih.gov/refseq/), 31 Geoemydidae species mitogenomes were downloaded from GenBank and incorporated in the dataset for comparative analysis (Table S1). The genome size and comparative analysis of nucleotide composition were calculated using MEGA6.0 (Tamura, Stecher, Peterson, Filipski, & Kumar, 2013). The direction and arrangements of each gene were also checked through MITOS online server. The overlapping regions and intergenic spacers between genes were counted manually in Microsoft Excel. The start and stop codons of PCGs were checked through the Open Reading Frame Finder (https://www.ncbi.nlm.nih.gov/orffinder/) web tool. The comparative analysis of relative synonymous codon usage (RSCU), relative abundance of amino acids, and codons distribution were calculated using MEGA6.0. The pairwise test of the synonymous (Ks) and nonsynonymous (Ka) substitutions were calculated between *Pangshura* and other Geoemydids using DnaSPv5.0 (Librado & Rozas, 2009). The tRNA genes were verified in MITOS online server, tRNAscan‐SE Search Server 2.0 (http://lowelab.ucsc.edu/tRNAscan-SE/) and ARWEN 1.2 with the default settings (Laslett & Canbäck, 2008; Lowe & Chan, 2016). The base composition of all stems (DHU, acceptor, TѱC, anticodon) were examined manually to distinguish the Watson‐Crick, wobble, and mismatched base pairing. The tandem repeats in the CR were predicted by the online Tandem Repeats Finder web tool (https://tandem.bu.edu/trf/trf.html; Benson, 1999). The base composition skew was calculated as described earlier: AT‐skew = [A − T]/[A + T], GC‐skew = [G − C]/[G + C] (Perna & Kocher, 1995).

### 2.5 Phylogenetic analysis

To assess the phylogenetic relationship, the 13 PCGs of 37 mitogenomes (32 Geoemydidae species and five species from other taxonomic lineages) were aligned individually by codons using MAFFT algorithm in TranslatorX with L‐INS‐i strategy with GBlocks parameters and default settings (Abascal, Zardoya, & Telford, 2010). The database sequence of *Chelus fimbriata* (accession no. HQ172156) under family Chelidae (suborder: Pleurodira) was used as an out‐group in both ML and BA phylogenetic analysis. The dataset of all PCGs was concatenated (10,647 bp) using SequenceMatrix v1.7.84537 (Vaidya, Lohman, & Meier, 2010). The aligned dataset was further submitted to the web service, TreeBASE version 2 (Piel et al., 2009) and made publicly available (http://purl.org/phylo/treebase/phylows/study/TB2:S24607). The model test and phylogenetic analysis were performed at the CIPRES Science Gateway V. 3.3 (Miller et al., 2015). Six models were estimated and tested separately through PartitionFinder 2 (Lanfear, Frandsen, Wright, Senfeld, & Calcott, 2016; Table S2). The maximum‐likelihood (ML) tree was constructed using IQ‐Tree web server (Trifinopoulos, Nguyen, von Haeseler, & Minh, 2016) with the bootstrap support for each branch nodes were fixed with 1,000 replicates. The Bayesian analysis (BA) was performed through Mr. Bayes 3.1.2 (Ronquist & Huelsenbeck, 2003). The metropolis‐coupled Markov Chain Monte Carlo (MCMC) was run for 100,000,000 generations with sampling at every 100th generation and 25% of samples were discarded as burn‐in. Both ML and BA tree were further processed in iTOL v4. Interactive Tree of Life online tool for better representation (Letunic & Bork, 2007).

## 3 RESULTS AND DISCUSSION

### 3.1 Mitogenome structure and organization

**Figure 1:**
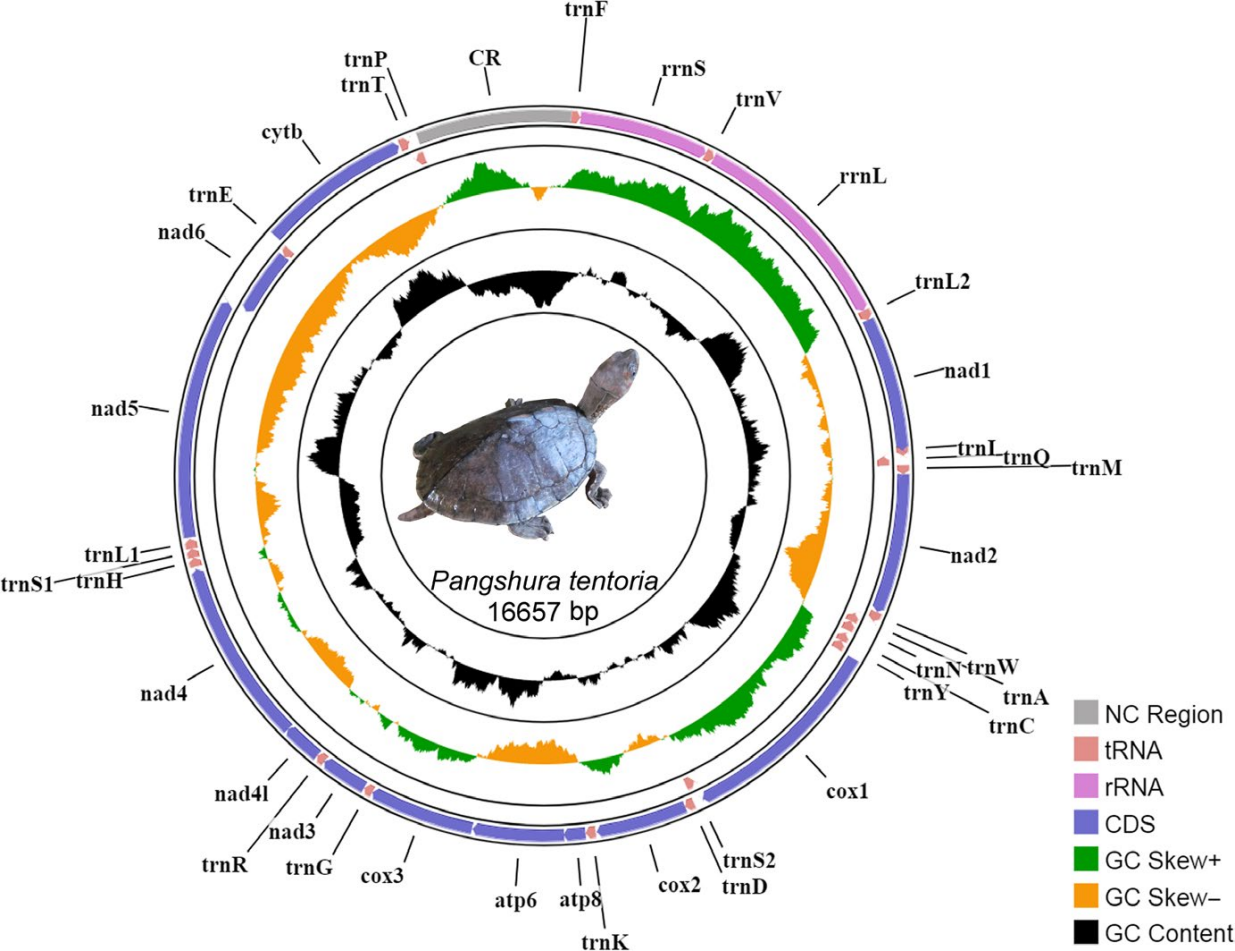
The mitochondrial genome of *P. tentoria*. Direction of gene transcription is indicated by arrows. Protein‐coding genes are shown as violet arrows, rRNA genes as purple arrows, tRNA genes as pink arrows and noncoding region as gray rectangle. The GC content is plotted using a black sliding window, GC‐skew is plotted using green and orange color sliding window as the deviation from the average in the complete mitogenome. The figure was drawn using CGView online server (http://stothard.afns.ualberta.ca/cgview_server/) with default parameters. The Species photographs were taken by the first authors (S.K.) by using Nikon D3100 and edited manually in Adobe Photoshop CS 8.0

In this study, the complete mitogenome (16,657 bp) of Indian Tent Turtle, *P. tentoria* was determined (GenBank accession no. MH795989). The mitogenome was encoded by 37 genes, including 13 PCGs, 22 tRNAs, two rRNAs, and a major noncoding CR. Among these, 28 genes (12 PCGs, 14 tRNAs, and two rRNAs) were located on the majority strand and the remaining genes (NADH dehydrogenase subunit 6 and eight tRNAs) were located on the minority strand (Table 1, Figure 1). In other Geoemydid species, the locations of 37 genes are similar to *P. tentoria* in both majority and minority strands (Table S3). The study depicted the gene arrangements of *P. tentoria* were the same as in the typical vertebrate gene arrangement (Anderson et al., 1982). The nucleotide composition of *P. tentoria* mitogenome was biased toward A + T (59.44%). The A + T composition of PCGs, tRNAs, rRNAs, and CR was 58.52%, 60.28%, 58.86%, and 66.06%, respectively. In other Geoemydid species, the A + T composition was also similar to *P. tentoria* and biased toward A + T with a variable frequency ranging from 58.12% (*B. trivittata*) to 62.52% (*H. depressa*). The AT‐skew was 0.120, and GC‐skew was −0.331 in the *P. tentoria* mitogenome. The comparative analysis revealed that the AT‐skew varied from 0.100 (*C. aurocapitata* and *N. platynota*) to 0.156 (*B. trivittata*), and GC‐skew varied from −0.366 (*B. trivittata*) to −0.320 (*C. dentata*) in other Geoemydid species (Table 2).

**Table 1:** List of annotated mitochondrial genes of *Pangshura tentoria*

Gene	Direction	Location	Size (bp)	Anticodon	Start codon	Stop codon	Intergenic nucleotides
trnF	+	203–271	69	GAA	.	.	0
rrnS	+	272–1235	964	.	.	.	0
trnV	+	1236–1304	69	TAC	.	.	−1
rrnL	+	1304–2901	1,598	.	.	.	1
trnL2	+	2903–2978	76	TAA	.	.	0
nad1	+	2979–3938	960	.	ATG	(A)	8
trnI	+	3947–4017	71	GAT	.	.	−1
trnQ	−	4017–4087	71	TTG	.	.	−1
trnM	+	4087–4155	69	CAT	.	.	0
nad2	+	4156–5190	1,035	.	ATG	(A)	4
trnW	+	5195–5267	73	TCA	.	.	1
trnA	−	5269–5337	69	TGC	.	.	1
trnN	−	5339–5412	74	GTT	.	.	27
trnC	−	5440–5505	66	GCA	.	.	0
trnY	−	5506–5577	72	GTA	.	.	1
cox1	+	5579–7114	1,536	.	GTG	(A)	3
trnS2	−	7118–7188	71	GCT	.	.	0
trnD	+	7189–7258	70	GTC	.	.	0
cox2	+	7259–7936	678	.	ATG	(T)	10
trnK	+	7947–8020	74	TTT	.	.	1
atp8	+	8022–8183	162	.	ATG	(A)	−4
atp6	+	8180–8857	678	.	ATG	(A)	5
cox3	+	8863–9645	783	.	ATG	(TA)	1
trnG	+	9647–9714	68	TCC	.	.	1
nad3	+	9716–10064	354	.	ATG	(T)	1
trnR	+	10066–10134	69	TCG	.	.	0
nad4l	+	10135–10428	294	.	ATG	(TAA)	−4
nad4	+	10425–11795	1,371	.	ATG	(A)	20
trnH	+	11816–11884	69	GTG	.	.	0
trnS1	+	11885–11951	67	TGA	.	.	−1
trnL1	+	11951–12023	73	TAG	.	.	12
nad5	+	12036–13823	1,788	.	ATA	(A)	4
nad6	−	13828–14349	522	.	ATG	(AGA)	0
trnE	−	14350–14417	68	TTC	.	.	4
cytb	+	14422–15555	1,134	.	ATG	(A)	10
trnT	+	15566–15637	72	TGT	.	.	0
trnP	−	15638–15708	71	TGG	.	.	0
A + T‐rich Region		15709–16657 1–202	1,151	.	.	−	.

Note

Direction of genes are denoted by (+) for majority and (−) for minority strands. The (−) value in intergenic nucleotides column represent the overlapping regions between the genes.

**Table 2:** Nucleotide composition of the mitochondrial genome in different Geoemydid turtle's mtDNA

Species	Size (bp)	A%	T%	G%	C%	A + T%	AT‐skew	GC‐skew
Complete mitogenome								
*Pangshura tentoria*	16,657	33.30	26.13	13.54	27.00	59.44	0.120	−0.331
*Batagur trivittata*	16,463	33.60	24.52	13.25	28.62	58.12	0.156	−0.366
*Cuora amboinensis*	16,708	33.82	26.74	13.05	26.36	60.57	0.116	−0.337
*C. aurocapitata*	16,890	33.56	27.41	13.04	25.97	60.98	0.100	−0.331
*C. bourreti*	16,649	33.90	26.84	13.05	26.19	60.75	0.116	−0.334
*C. flavomarginata*	16,721	33.99	27.67	12.80	25.52	61.67	0.102	−0.331
*C. galbinifrons*	17,244	34.12	27.58	12.47	25.81	61.70	0.106	−0.348
*C. mouhotii*	16,837	34.03	27.33	12.81	25.81	61.37	0.109	−0.336
*C. pani*	16,922	33.67	27.43	13.00	25.89	61.10	0.102	−0.331
*C. picturata*	16,623	33.95	26.89	13.00	26.14	60.85	0.116	−0.335
*C. trifasciata*	16,675	33.84	26.83	13.12	26.18	60.68	0.115	−0.332
*Cyclemys atripons*	16,500	34.40	27.20	13.01	25.36	61.62	0.117	−0.321
*C. dentata*	16,484	34.28	27.22	13.08	25.41	61.50	0.114	−0.320
*C. oldhami*	16,656	34.35	26.83	13.10	25.71	61.18	0.122	−0.324
*C. pulchristriata*	16,527	34.38	27.19	12.98	25.43	61.57	0.116	−0.324
*C. tcheponensis*	16,593	34.20	26.77	13.19	25.83	60.97	0.121	−0.323
*Heosemys annandalii*	16,604	35.14	26.71	12.27	25.87	61.85	0.136	−0.356
*H. depressa*	16,773	35.00	27.52	12.53	24.93	62.52	0.119	−0.330
*H. grandis*	16,581	34.70	27.67	12.52	25.09	62.38	0.112	−0.334
*Mauremys annamensis*	16,844	33.70	26.85	13.04	26.38	60.56	0.113	−0.338
*M. caspica*	16,741	34.04	27.17	12.91	25.87	61.21	0.112	−0.334
*M. japonica*	16,443	34.02	26.45	13.01	26.50	60.48	0.125	−0.341
*M. leprosa*	17,066	34.41	27.48	12.43	25.66	61.90	0.111	−0.347
*M. megalocephala*	16,783	34.05	27.20	12.81	25.92	61.25	0.111	−0.338
*M. mutica*	16,609	33.81	26.50	13.17	26.49	60.32	0.121	−0.335
*M. nigricans*	16,779	34.07	26.85	12.96	26.09	60.93	0.118	−0.336
*M. reevesii*	16,576	33.99	26.62	12.94	26.44	60.61	0.121	−0.342
*M. rivulata*	16,766	34.31	26.91	12.94	25.83	61.22	0.120	−0.332
*M. sinensis*	16,461	33.81	26.20	13.17	26.79	60.02	0.126	−0.340
*Notochelys platynota*	16,981	34.39	28.10	12.24	25.25	62.49	0.100	−0.347
*Sacalia bealei*	16,561	34.18	26.86	13.06	25.88	61.04	0.119	−0.329
*S. quadriocellata*	16,816	34.13	26.75	13.16	25.94	60.88	0.121	−0.326
Protein‐coding genes (PCGs)								
*Pangshura tentoria*	11,295	30.78	27.73	13.51	27.96	58.52	0.052	−0.348
*Batagur trivittata*	11,379	31.37	26.07	13.05	29.49	57.44	0.092	−0.386
*Cuora amboinensis*	11,397	31.44	28.05	13.07	27.42	59.49	0.057	−0.354
*C. aurocapitata*	11,373	31.18	28.11	13.35	27.33	59.30	0.051	−0.343
*C. bourreti*	11,394	31.44	28.41	13.23	26.90	59.86	0.050	−0.340
*C. flavomarginata*	11,377	31.41	29.10	13.03	26.44	60.51	0.038	−0.339
*C. galbinifrons*	11,399	31.51	28.52	13.13	26.83	60.03	0.049	−0.342
*C. mouhotii*	11,387	31.57	28.74	13.13	26.53	60.32	0.047	−0.337
*C. pani*	11,393	31.10	28.28	13.29	27.30	59.39	0.047	−0.345
*C. picturata*	11,395	31.51	28.52	13.17	26.79	60.03	0.049	−0.340
*C. trifasciata*	11,382	31.28	28.31	13.31	27.08	59.60	0.049	−0.341
*Cyclemys atripons*	11,387	31.88	29.16	13.05	25.88	61.05	0.044	−0.329
*C. dentata*	11,376	31.82	29.16	13.09	25.91	60.98	0.043	−0.328
*C. oldhami*	11,370	31.34	28.78	13.35	26.50	60.13	0.042	−0.329
*C. pulchristriata*	11,380	31.76	29.04	13.06	26.12	60.80	0.044	−0.333
*C. tcheponensis*	11,377	31.33	28.80	13.41	26.44	60.13	0.042	−0.327
*Heosemys annandalii*	11,380	32.65	28.31	12.12	26.90	60.96	0.071	−0.378
*H. depressa*	11,382	32.26	29.24	12.77	25.71	61.50	0.048	−0.336
*H. grandis*	11,379	32.56	29.39	12.38	25.65	61.96	0.051	−0.348
*Mauremys annamensis*	11,391	31.34	28.05	13.15	27.44	59.39	0.055	−0.351
*M. caspica*	11,382	31.70	28.40	13.00	26.88	60.11	0.054	−0.348
*M. japonica*	11,385	31.87	28.18	12.84	27.09	60.06	0.061	−0.356
*M. leprosa*	11,382	31.92	28.73	12.89	26.43	60.66	0.052	−0.344
*M. megalocephala*	11,385	31.62	28.49	13.02	26.85	60.11	0.052	−0.346
*M. mutica*	11,392	31.43	27.94	13.29	27.33	59.37	0.058	−0.345
*M. nigricans*	11,382	31.50	28.14	13.18	27.15	59.65	0.056	−0.346
*M. reevesii*	11,377	31.81	28.02	13.04	27.11	59.84	0.063	−0.350
*M. rivulata*	11,382	31.68	28.41	13.03	26.86	60.09	0.054	−0.346
*M. sinensis*	11,395	31.68	27.86	13.04	27.40	59.54	0.064	−0.354
*Notochelys platynota*	11,398	32.12	29.47	12.50	25.89	61.60	0.043	−0.348
*Sacalia bealei*	11,373	31.82	28.57	12.92	26.67	60.39	0.053	−0.347
*S. quadriocellata*	11,366	31.76	28.56	12.96	26.70	60.32	0.052	−0.346
tRNA genes								
*Pangshura tentoria*	1,551	30.94	29.33	20.95	18.76	60.28	0.026	0.055
*Batagur trivittata*	1,551	30.75	29.27	20.88	19.08	60.02	0.024	0.045
*Cuora amboinensis*	1,608	32.46	30.09	19.21	18.22	62.56	0.037	0.026
*C. aurocapitata*	1,796	32.01	30.23	19.04	18.70	62.24	0.028	0.008
*C. bourreti*	1,553	32.13	29.62	19.63	18.60	61.75	0.040	0.026
*C. flavomarginata*	1,553	32.38	30.00	19.51	18.09	62.39	0.038	0.037
*C. galbinifrons*	1,552	32.02	29.25	19.78	18.94	61.27	0.045	0.021
*C. mouhotii*	1,552	32.02	29.83	19.65	18.49	61.85	0.035	0.030
*C. pani*	1,554	32.23	29.60	19.49	18.66	61.84	0.042	0.021
*C. picturata*	1,553	32.19	29.55	19.63	18.60	61.75	0.042	0.026
*C. trifasciata*	1,553	31.74	29.74	19.76	18.73	61.49	0.032	0.026
*Cyclemys atripons*	1,551	32.10	30.10	19.66	18.11	62.21	0.032	0.040
*C. dentata*	1,548	32.55	30.03	19.25	18.15	62.59	0.040	0.029
*C. oldhami*	1,551	32.62	30.04	19.21	18.11	62.66	0.041	0.029
*C. pulchristriata*	1,551	32.10	30.36	19.66	17.85	62.47	0.027	0.048
*C. tcheponensis*	1,606	33.37	30.57	18.67	17.37	63.94	0.043	0.036
*Heosemys annandalii*	1,550	32.25	29.87	19.35	18.51	62.12	0.038	0.022
*H. depressa*	1,549	31.76	29.69	20.07	18.46	61.45	0.033	0.041
*H. grandis*	1,549	32.27	29.82	19.49	18.39	62.10	0.039	0.028
*Mauremys annamensis*	1,496	32.41	30.08	19.18	18.31	62.50	0.037	0.023
*M. caspica*	1,554	32.17	29.66	19.62	18.53	61.84	0.040	0.028
*M. japonica*	1,557	32.24	29.60	19.52	18.62	61.84	0.042	0.023
*M. leprosa*	1,552	32.73	29.51	19.13	18.62	62.24	0.051	0.013
*M. megalocephala*	1,554	32.36	29.72	19.49	18.40	62.09	0.042	0.028
*M. mutica*	1,553	32.58	30.13	19.18	18.09	62.71	0.039	0.029
*M. nigricans*	1,555	32.60	29.58	19.35	18.45	62.18	0.048	0.023
*M. reevesii*	1,547	32.25	29.99	19.52	18.22	62.24	0.036	0.034
*M. rivulata*	1,551	32.17	29.91	19.47	18.43	62.08	0.036	0.027
*M. sinensis*	1,555	32.15	29.71	19.67	18.45	61.86	0.039	0.032
*Notochelys platynota*	1,551	32.49	29.98	19.27	18.24	62.47	0.040	0.027
*Sacalia bealei*	1,549	32.08	29.89	20.01	18.01	61.97	0.035	0.052
*S. quadriocellata*	1,548	31.97	29.84	20.09	18.08	61.82	0.034	0.052
rRNA genes								
*Pangshura tentoria*	2,562	37.23	21.62	17.36	23.77	58.86	0.265	−0.155
*Batagur trivittata*	2,568	37.26	20.52	17.44	24.76	57.78	0.289	−0.173
*Cuora amboinensis*	2,572	37.67	21.38	16.95	23.98	59.05	0.275	−0.171
*C. aurocapitata*	2,577	37.91	21.57	16.99	23.51	59.48	0.274	−0.160
*C. bourreti*	2,571	38.11	21.31	16.60	23.95	59.43	0.282	−0.181
*C. flavomarginata*	2,562	38.09	22.24	16.66	22.98	60.34	0.262	−0.159
*C. galbinifrons*	2,571	38.23	21.89	16.25	23.60	60.13	0.271	−0.184
*C. mouhotii*	2,570	38.13	21.67	16.65	23.54	59.80	0.275	−0.171
*C. pani*	2,568	37.96	21.53	16.97	23.52	59.50	0.276	−0.161
*C. picturata*	2,553	38.22	21.38	16.56	23.81	59.61	0.282	−0.179
*C. trifasciata*	2,568	38.04	21.30	16.78	23.87	59.34	0.282	−0.174
*Cyclemys atripons*	2,561	38.73	21.98	16.39	22.88	60.71	0.275	−0.165
*C. dentata*	2,565	38.55	21.94	16.56	22.92	60.50	0.274	−0.160
*C. oldhami*	2,569	38.45	21.44	16.77	23.31	59.90	0.283	−0.163
*C. pulchristriata*	2,564	38.72	21.95	16.41	22.89	60.68	0.276	−0.164
*C. tcheponensis*	2,576	38.31	21.35	16.73	23.60	59.66	0.284	−0.170
*Heosemys annandalii*	2,563	39.32	22.27	16.07	22.31	61.60	0.276	−0.162
*H. depressa*	2,565	38.71	22.84	16.21	22.22	61.55	0.257	−0.156
*H. grandis*	2,566	38.73	22.36	16.32	22.56	61.10	0.267	−0.160
*Mauremys annamensis*	2,715	37.56	21.76	16.64	24.01	59.33	0.266	−0.181
*M. caspica*	2,568	37.88	21.65	16.78	23.67	59.54	0.272	−0.170
*M. japonica*	2,570	37.93	21.78	16.69	23.57	59.72	0.270	−0.171
*M. leprosa*	2,567	38.05	21.97	16.67	23.29	60.03	0.268	−0.165
*M. megalocephala*	2,574	37.91	21.91	16.55	23.62	59.82	0.267	−0.176
*M. mutica*	2,568	37.65	21.80	16.93	23.59	59.46	0.266	−0.164
*M. nigricans*	2,570	38.21	21.59	16.34	23.85	59.80	0.277	−0.186
*M. reevesii*	2,573	37.89	21.99	16.51	23.59	59.89	0.265	−0.176
*M. rivulata*	2,567	37.78	21.58	16.86	23.76	59.36	0.272	−0.169
*M. sinensis*	2,570	37.50	21.43	16.88	24.16	58.94	0.272	−0.177
*Notochelys platynota*	2,573	38.55	22.46	16.28	22.69	61.01	0.263	−0.164
*Sacalia bealei*	2,574	38.11	21.87	16.70	23.31	59.98	0.270	−0.165
*S. quadriocellata*	2,859	37.46	21.93	16.96	23.64	59.39	0.261	−0.164
Control regions								
*Pangshura tentoria*	949	32.03	34.03	12.96	20.96	66.06	−0.030	−0.236
*Batagur trivittata*	947	31.67	33.26	12.98	22.06	64.94	−0.024	−0.259
*Cuora amboinensis*	1,182	33.16	40.27	10.74	15.82	73.43	−0.096	−0.191
*C. aurocapitata*	1,379	33.06	43.65	9.35	13.92	76.72	−0.138	−0.196
*C. bourreti*	1,128	32.53	39.53	10.72	17.19	72.07	−0.097	−0.231
*C. flavomarginata*	1,207	33.88	40.84	9.61	15.65	74.73	−0.093	−0.239
*C. galbinifrons*	1,722	34.90	41.28	6.79	17.01	76.19	−0.083	−0.429
*C. mouhotii*	1,316	33.66	39.81	8.81	17.70	73.48	−0.083	−0.335
*C. pani*	1,402	33.16	44.15	8.98	13.69	77.31	−0.142	−0.207
*C. picturata*	1,120	32.50	39.10	10.71	17.67	71.60	−0.092	−0.245
*C. trifasciata*	1,156	33.91	39.79	10.03	16.26	73.70	−0.079	−0.236
*Cyclemys atripons*	981	34.76	34.76	12.13	18.34	69.52	0	−0.204
*C. dentata*	973	33.09	35.25	12.84	18.80	68.34	−0.031	−0.188
*C. oldhami*	1,149	37.94	34.72	10.79	16.53	72.67	0.044	−0.210
*C. pulchristriata*	1,016	35.62	35.62	11.61	17.12	71.25	0	−0.191
*C. tcheponensis*	1,073	36.25	34.20	11.64	17.89	70.45	0.029	−0.211
*Heosemys annandalii*	1,095	34.79	36.43	10.59	18.17	71.23	−0.023	−0.263
*H. depressa*	1,262	37.71	37.55	9.35	15.37	75.27	0.002	−0.243
*H. grandis*	1,072	31.34	39.08	11.38	18.19	70.42	−0.109	−0.230
*Mauremys annamensis*	1,176	32.48	40.39	10.54	16.58	72.87	−0.108	−0.222
*M. caspica*	1,223	33.44	41.29	9.89	15.37	74.73	−0.105	−0.216
*M. japonica*	914	32.38	33.80	13.01	20.78	66.19	−0.021	−0.229
*M. leprosa*	1,615	34.61	38.76	7.55	19.07	73.37	−0.056	−0.432
*M. megalocephala*	1,254	33.97	40.19	9.56	16.26	74.16	−0.083	−0.259
*M. mutica*	1,071	32.49	37.44	11.20	18.86	69.93	−0.070	−0.254
*M. nigricans*	1,253	35.27	38.54	10.05	16.12	73.82	−0.044	−0.231
*M. reevesii*	1,072	34.32	34.32	11.28	20.05	68.65	0	−0.279
*M. rivulata*	1,252	37.53	37.22	10.06	15.17	74.76	0.004	−0.202
*M. sinensis*	935	31.65	34.75	12.40	21.17	66.41	−0.046	−0.261
*Notochelys platynota*	1,457	32.18	40.28	8.30	19.21	72.47	−0.111	−0.396
*Sacalia bealei*	1,048	32.72	36.54	11.92	18.79	69.27	−0.055	−0.223
*S. quadriocellata*	1,046	33.26	36.23	11.56	18.92	69.50	−0.042	−0.241

Note

The A + T biases of whole mitogenome, protein‐coding genes, tRNA, rRNA, and control regions were calculated by AT‐skew = (A‐T)/(A + T) and GC‐skew = (G‐C)/(G + C), respectively.

### 3.2 Overlapping and intergenic spacer regions

Six overlapping regions with a total length of 12 bp were identified in *P. tentoria* mitogenome. These regions varied in length from 1 to 4 bp with the longest overlapping region presented between NADH dehydrogenase subunit 4 L (*nad4l*) and NADH dehydrogenase subunit 4 (*nad4*) as well as in between ATP synthase F0 subunit 8 (*atp8*) and ATP synthase F0 subunit 6 (*atp6*). In other Geoemydid species, the number of overlapping regions varied from four to six with a length variation 18 bp (*C. dentata*) to 94 bp (*M. leprosa*) with the longest overlapping region (67 bp) located between tRNA‐Proline (*trnP*) and CR of *M. leprosa*. The intergenic spacers in *P. tentoria* mitogenome were spread over 19 regions and ranged from 1 to 27 bp with a total length of 115 bp. The longest spacer (27 bp) was observed between tRNA‐Asparagine (*trnN*) and tRNA‐Cysteine (*trnC*; Table 1). In other Geoemydid species, the longest intergenic spacer of 29 bp was present between tRNA‐Asparagine (*trnN*) and tRNA‐Cysteine (*trnC*) of *N. platynota* (Table S4).

### 3.3 Protein‐coding genes

The total length of PCGs was 11,295 bp in *P. tentoria*, which represents 67.8% of complete mitogenome. The nucleotide composition, AT‐skew and GC‐skew of PCGs in comparable Geoemydid species, is outlined in Table 2. The A + T composition was 58.52% in PCGs of *P. tentoria*. In other species, The A + T composition varied from 57.44% (*B. trivittata*) to 61.96% (*H. grandis*). The AT‐skew of PCGs was 0.052, and GC‐skew was −0.348 in *P. tentoria*. The AT‐skew in other Geoemydid species varied from 0.038 (*C. flavomarginata*) to 0.092 (*B. trivittata*), and GC‐skew varied from −0.386 (*B. trivittata*) to −0.327 (*C. tcheponensis*). Most of the PCGs of *P. tentoria* started with an ATG initiation codon, similar to other Geoemydid species. The “GTG” initiation codon was observed in Cytochrome oxidase subunit 1 (*cox1*) gene of *P. tentoria* and other Geoemydid species except *C. dentata*. The “ATA” initiation codon was observed in NADH dehydrogenase subunit 5 (*nad5*) of *P. tentoria*; NADH dehydrogenase subunit 2 (*nad2*) in *B. trivittata* and *C. amboinensis*; Cytochrome *b* (*cytb*) in *C. aurocapitata* and *M. reevesii*. Further, the “ATT” was observed only in *nad6* of *M. annamensis*. The 11 PCGs of *P. tentoria* used incomplete termination codons with few exceptions like “TAA” for *nad4l* and “AGA” for *nad6* (Table S5). The comparative analysis of all the Geoemydid species revealed “TAA” termination codon for almost all PCGs except 14 Geoemydid species which used “TAG” termination codon for six PCGs: Cytochrome oxidase subunit 2 (*cox2*), NADH dehydrogenase subunit 6 (*nad1*), *nad2*, NADH dehydrogenase subunit 3 (*nad3*), *nad4*, and *nad6*. Further, the “AGA” termination codon was used by two PCGs (*nad3*, and *nad6*). “AGG” termination codon was used by *cox1* for all the species and *nad6* in most of the species except, *B. trivittata*, *M. reevesii*, *M. sinensis*, and *S. quadriocellata*. The incomplete TA(G) termination codon was used by Cytochrome oxidase subunit 3 (*cox3*), *nad5*, *cytb*, and *nad1*. The incomplete termination codon “T” was also observed in *nad2*, *cox3*, *nad6*, and *cytb* (Table S5).

### 3.4 Relative synonymous codon usage

The RSCU analysis revealed a maximum abundance of alanine, isoleucine, leucine, and threonine in the PCGs of *P. tentoria*, whereas Arginine, Aspartic Acid, Cysteine, and Lysine were less abundant (Figure S4). In other Geoemydid species, maximum abundance of alanine, Asparagine, isoleucine, leucine, serine, and threonine was observed, and Arginine, Aspartic Acid, Cysteine, and Lysine were less abundant. The RSCU analysis of *P. tentoria* also indicated the major proportion of codons bearing Cytosine (C) or Guanine (G) in the third position rather than Adenine (A) and Thymine (T). The relative usage of the AAC and GAC codon was more, compared to the AAT and GAT codon in the case of Asparagine and Aspartic Acid respectively. This same usage was more or less observed in other Geoemydid species. The comparative RSCU analysis indicated a clear fall in the frequency of TTG codon in leucine (Leu) in *B. trivittata*, *C. amboinensis*, *C. aurocapitata*, *C. flavomarginata*, and *C. galbinifrons* (Figure S5). Further, the noticeable fall in the frequency of TCG codon in serine (Ser) was observed in *C. oldhamii*, *H. annandalii*, *M. reevesii*, *N. platynota* and ACG codon in threonine (Thr) was observed in *P. tentoria*. Codon distribution per thousand codon (CDsPT) values for all the amino acids showed the same result and the maximum CDsPT value for leucine was observed in *P. tentoria* (165.5) and minimum value was observed in *S. quadriocellata* (117.7; Figure S6).

### 3.5 Synonymous and nonsynonymous substitutions

Darwinian selection plays an important role behind species divergence (Mikkelsen et al., 2005). The utility of mitogenomes for detecting positive selection that acts on PCGs can shed light on natural selection which may affect protein function (Bloom, Labthavikul, Otey, & Arnold, 2006; Hirsh & Fraser, 2001). These pairwise tests of the Ks and Ka substitutions were evidence of the adaptive evolution in vertebrates and other species (Montoya‐Burgos, 2011; Yang & Nielsen, 2000). It was stated that, the Ka/Ks > 1 evidenced for positive selection, Ka/Ks = 1 for neutrality, and Ka/Ks < 1 for negative selection (Chakraborty et al., 2018). To explore evolutionary rates, Ka/Ks substitutions were calculated for *P. tentoria* and other Geoemydid species mitogenomes. The Ka/Ks values of 13 PCGs varied from 0.006 (between *P. tentoria* and *B. trivittata* in *cox1*) to 0.549 (between *P. tentoria* and *H. annandalii* in *nad6*). All PCGs showed Ka/Ks values <1 which suggested a strong negative selection among all Geoemydid species which intended natural selection. The percentage of Ka/Ks variation was highest in *nad6*, ranging from 0.189 to 0.549, which suggest the minimum selective pressure in *nad6* gene. As Ka/Ks ratio is least in *cox1*, ranging from 0.006 to 0.015, this PCG is recognized under most selective pressure. Among all the species pair, *P. tentoria* and *B. trivittata* showed least Ka/Ks value (0.006 in *cox1*) as compared to other species pairs, implying a closer phylogenetic relationship between these two species. The Ka/Ks ratio of all the PCGs follows the order: *cox1* < *cox3* < *cox2* < *cytb* < *atp6* < *nad3* < *nad5* < *atp8* < *nad4* < *nad4l* < *nad2* < *nad1* < *nad6* (Figure S7). Thus, comparative analysis of Ka/Ks in Geoemydid species mitogenomes will help to understand the natural selection and evolution of species.

### 3.6 Transfer RNAs and ribosomal RNAs

The wobble base pairing is a unique characteristic of RNA secondary structure and often replaces the GC or AT base pairs due to the thermodynamic stability (Yang & Bielawski, 2000). RNA‐binding proteins bind to G‐U sites and differ from Watson–Crick base pairs (Crick, 1966). Hence, the characteristics of tRNAs secondary structures are essential for understanding the functional role of the mitogenomes (Varani & McClain, 2000). The total length of 22 tRNAs of *P. tentoria* mitogenome was 1,551 bp ranging from 67 bp to 76 bp with a total of 60.28% A + T content. In other Geoemydid species, total length of tRNAs varied from 1,496 bp (*M. annamensis*) to 1,796 bp (*C. aurocapitata*). The A + T content of other Geoemydid species varied from 60.02% (*B. trivittata*) to 63.94% (*C. tcheponensis*). The AT‐skew and GC‐skew of tRNA genes of *P. tentoria* were 0.026 and 0.055, respectively. The AT‐skew of other Geoemydid species varied from 0.024 (*B. trivittata*) to 0.051 (*M. leprosa*) and GC‐skew from 0.008 (*C. aurocapitata*) to 0.052 (*S. bealei* and *S. quadriocellata*; Table 2). Among all 22 tRNA genes, 14 were on majority strand and remaining eight (*trnQ*, *trnA*, *trnN*, *trnC*, *trnY*, *trnS2*, *trnE*, and *trnP*) on the minority strand (Table S3). The anticodons of all tRNAs genes were similar in all Geoemydid species including *P. tentoria* (Table S6). The tRNAs were folded into classic clover‐leaf secondary structures, except for *trnS1*, which lacked the conventional DHU stem. It has been evidenced that, the unique arrangements “WANCY” in vertebrates, have an important role in the replacement function of the minority strand in mitogenomes (Satoh, Miya, Mabuchi, & Nishida, 2016). The similar tRNAs arrangements were also observed in *P. tentoria* mitogenome and other Geoemydid species. In the tRNA secondary structures of *P. tentoria*, the Watson–Crick base pairing were found in most of the positions (Figure S8). The highest changes of base pairing were observed in *trnQ*, while no changes were observed in *trnR* and *trnT*. Further, the wobble base pairing was observed in 11 tRNAs: in the acceptor stem of *trnA*, *trnQ*, *trnN*, *trnC*, *trnY*, *trnE*, *trnP*; in the TѱC stem of *trnQ*, *trnN*, *trnC*, *trnG*; in the anticodon stem of *trnL2*, *trnQ*, *trnN*, *trnS2*, *trnG*, *trnL1*, *trnE*, *trnP*; and in the DHU stem of *trnA*, *trnQ*, *trnN*, *trnY*, *trnS2*, *trnG*, *trnP* (Figure S8). The length of rRNA genes in *P. tentoria* was 2,562 bp and varied from 2,553 bp (*C. picturata*) to 2,859 bp (*S. quadriocellata*). The A + T composition of rRNA genes in *P. tentoria* was 58.86% and varied from 57.78% (*B. trivittata*) to 61.60% (*H. annandalii*). The AT‐skew and GC‐skew of *P. tentoria* rRNA genes were 0.265 and −0.155, respectively. In other Geoemydid species, The AT‐skew varied from 0.257 (*H. depressa*) to 0.289 (*B. trivittata*) and GC‐skew from −0.186 (*M. nigricans*) to −0.156 (*H. depressa*; Table 2).

### 3.7 Control regions (CRs)

**Figure 2:**
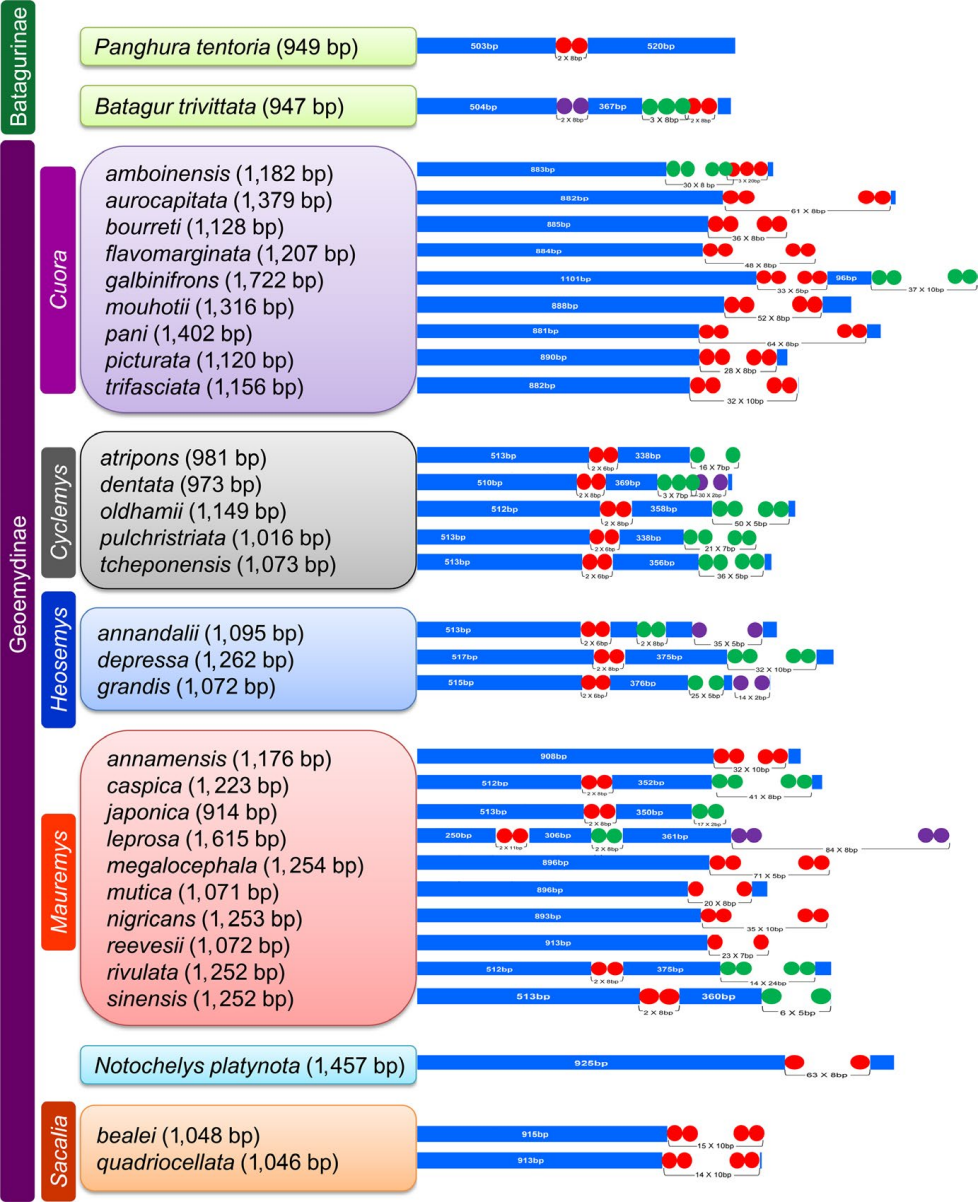
The structural organization of the control region of 32 Geoemydid species mitogenomes. The location and copy number of tandem repeats are shown by colored circles (Red, Green, and Violet). Nonrepeat regions are indicated by blue colored box with sequence size inside. The tandem repeats were predicted by the online Tandem Repeats Finder web tool (https://tandem.bu.edu/trf/trf.html) and edited manually in Adobe Photoshop CS 8.0. Color boxes indicate the species under respective taxonomic groups

The CR is typically allocated with three functional domains: the termination associated sequence (TAS), the central conserved (CD), and the conserved sequence block (CSB; Macey, Larson, Ananjeva, Fang, & Papenfuss, 1997). The CD domain with flexible numbers of tandem repeats (VNTRs) considered as the origin of the H‐strand transcription (Brown, Gadaleta, Pepe, Saccone, & Sbisà, 1986). These patterns of conserved sequences alter within different vertebrate groups, including turtles (Ruokonen & Kvist, 2002; Wang, Zhou, & Nie, 2011). The CR is also known for the initiation of replication in vertebrates, including Geoemydid species, and is located between *trnP* and *trnF* with a varying size (Bing, Fei, Yi, & Qing‐Wei, 2006; Zheng et al., 2013). The length of the CR of *P. tentoria* was 949 bp with 66.06% A + T composition (Table 2). In the other Geoemydid species, the length of CR varied from 914 bp (*M. japonica*) to 1,722 bp (*C. galbinifrons*) with AT composition ranges from 64.94% (*B. trivittata*) to 77.31% (*C. pani*). It is concluded that Adenine (A) composition is equal to Thymine (T) composition in *C. atripons*, *C. pulchristriata*, and *M. reevesii*. Adenine (A) composition is more as compared to Thymine (T) in *C. oldhamii*, *C. tcheponensis*, *H. depressa,* and *M. rivulata*. The other Geoemydid species have less Adenine (A) composition as compared to Thymine (T). The AT‐ and GC‐skew was negative, −0.030 and −0.236, respectively, in *P. tentoria*. The AT‐ and GC‐skew of other Geoemydid species ranges from −0.142 (*C. pani*) to 0.044 (*C. oldhamii*) and −0.432 (*M. leprosa*) to −0.188 (*C. dentata*), respectively. A single, tandem repeat of eight base pairs (TTCTCTTT) with two copy numbers was observed in *P. tentoria* (Figure 2). The numbers of tandem repeats are higher at the 3′ end of the CR in most of the studied Geoemydid species (Figure 2). Among all the Geoemydid species, 15 species had a single tandem repeat varied from 5 bp (*M. megalocephala*) to 10 bp (*C. trifasciata*, *M. annamensis*, *M. nigricans*). The comparative analysis revealed that, five species (*B. trivittata*, *C. dentata*, *H. annandalii*, *H. grandis*, and *M. leprosa*) were comprised of three different tandem repeats, while 11 species (*C. amboinensis*, *C. galbinifrons*, *C. atripons*, *C. oldhamii*, *C. pulchristriata*, *C. tcheponensis*, *H. depressa*, *M. caspica*, *M. japonica*, *M. rivulata*, and *M. sinensis*) were comprised of two different tandem repeats. Overall, the CR of Geoemydid species showed a specific sequence and structural feature, which was species‐specific and can be used as a molecular marker.

### 3.8 Phylogeny of Geoemydid mitogenomes

**Figure 3:**
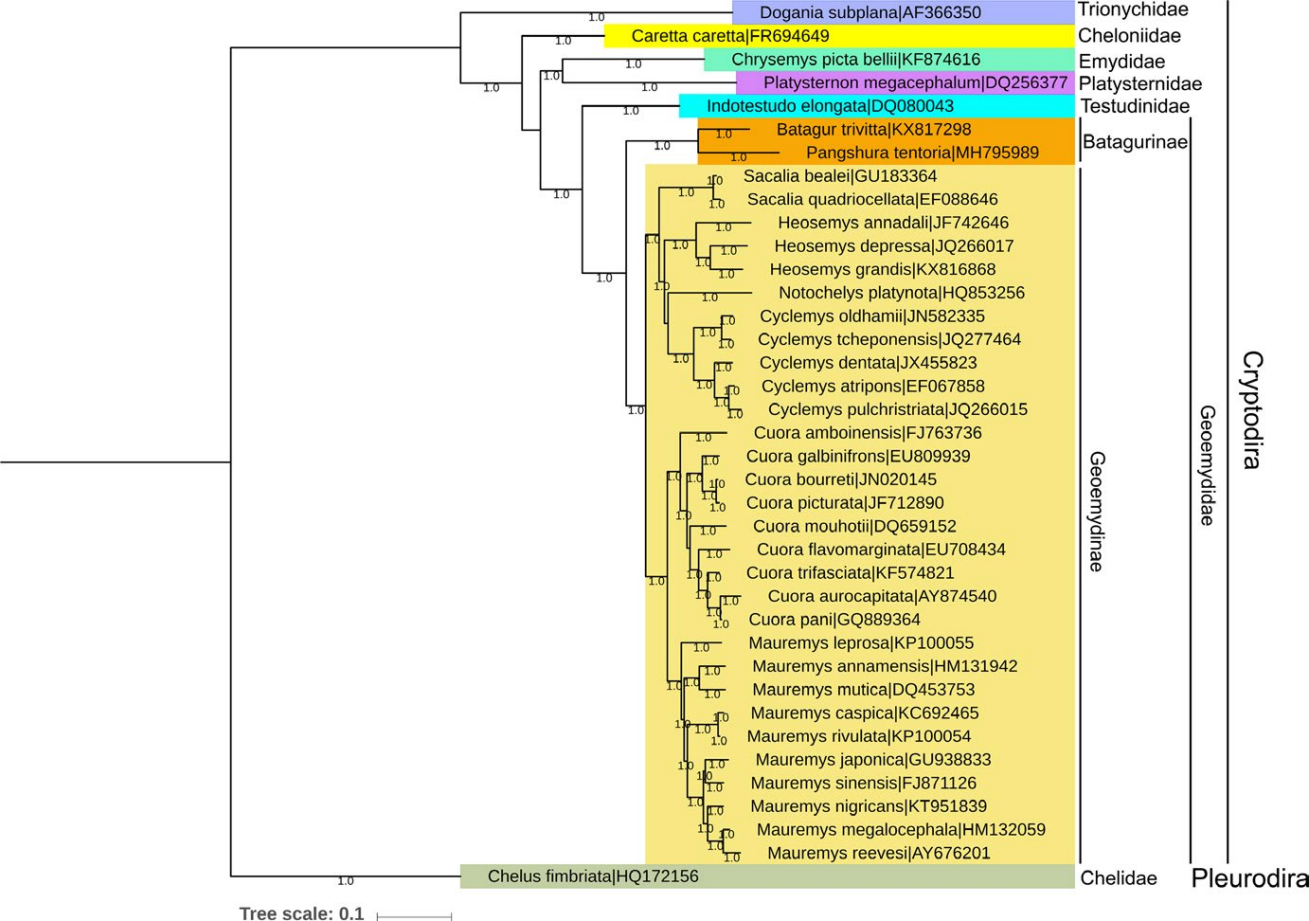
Bayesian (BA) phylogenetic tree based on the concatenated nucleotide sequences of 13 PCGs of 32 Geoemydid species showing the evolutionary relationship of *P. tentoria*. Species names and GenBank accession numbers are indicated within parentheses with each node. Color boxes indicate the species clustering under respective taxonomic groups. The BA tree is drawn to scale with posterior probability support values were indicated along with the branches. The BA tree was drawn in CIPRES Science Gateway V. 3.3 and edited with Itol v4 and Adobe Photoshop CS 8.0

Both mitochondrial and nuclear genes have been widely used for effective species identification and delimitation in Testudines (Fritz et al., 2008; Ihlow et al., 2016). However, to better understand evolutionary relationships and phylogeny within Testudines, datasets representing more taxa and loci are needed (Le et al., 2006). Comparative mitogenomic data have demonstrated utility in elucidating the phylogenetic relationships of turtles (Kundu, Kumar, Tyagi, et al., 2018; Li et al., 2017). The genus *Pangshura* was erected from *Batagur* and elevated as a distinct genus through morphological characteristics (Günther, 1864; Moll, 1986). Indeed, molecular studies with limited sampling supported *Pangshura* as a discrete monophyletic genus with four species (Spinks, Shaffer, Iverson, & McCord, 2004). Further, extensive taxon sampling of all species/subspecies using mitochondrial DNA corroborated the well‐supported monophyly of *Pangshura* (Shaffer, Meylan, & McKnight, 1997). Both the BA and ML phylogeny depicted similar topology with high posterior probability and bootstrap supports (Figures 3 and S9). All Geoemydid species were closely clustered and congruent with the previous evolutionary hypotheses (Guillon, Guéry, Hulin, & Girondot, 2012; Le, McCord, & Iverson, 2007). Our analysis confirms that *P. tentoria* shows a sister clade relationship with *Batagur trivittata* as described earlier. The genus *Mauremys*, *Cuora*, *Cyclemys*, *Heosemys*, *Sacalia*, and *Notochelys*, respectively, were reciprocally monophyletic with significant bootstrap supports. Further, the representative mitogenome sequences of other families/suborders, Testudinidae, Emydidae, https://www.ncbi.nlm.nih.gov/Taxonomy/Browser/wwwtax.cgi?mode=Tree%26xml:id=1579279%26lvl=3%26lin=f%26keep=1%26srchmode=1%26unlock, Cheloniidae, Trionychidae under suborder Cryptodira (hidden‐necked turtles) and Chelidae under suborder Pleurodira (side‐necked turtles) were clustered distinctly in both BA and ML phylogeny. The present mitogenomes study was able to generate a robust phylogeny and divergence time with high statistical values for each node and elucidate the relationship between *Pangshura* and other Geoemydid species. In addition, based on the jaw morphology, the family Geoemydidae was divided into two subfamilies Geoemydinae and Batagurinae (Gaffney & Meylan, 1988). Nevertheless, a single complete mitochondrial genome of Batagurinae taxa (*B. trivittata*) is available so far and the present study contributes the denovo assembly of *P. tentoria* mitogenome in the global database. We propose more taxon sampling of Batagurinae from different geographical locations, in the expectation that their mitogenomes will be useful to reconcile in‐depth phylogeny and evolutionary relationship.

## CONFLICT OF INTEREST

The authors declare that they have no competing interests.

## AUTHOR CONTRIBUTIONS

Shantanu Kundu, Vikas Kumar involved in conceptualization; Rajasree Chakraborty, Shantanu Kundu involved in data curation; Kaomud Tyagi, Shantanu Kundu involved in formal analysis; Kailash Chandra, Vikas Kumar involved in funding acquisition; Shantanu Kundu, Vikas Kumar involved in investigation; Shantanu Kundu, Rajasree Chakraborty involved in methodology; Kailash Chandra, Vikas Kumar involved in project administration; Kailash Chandra involved in resources; Shantanu Kundu, Kaomud Tyagi involved in software; Vikas Kumar, Kailash Chandra involved in supervision; Shantanu Kundu, Vikas Kumar involved in validation; Shantanu Kundu, Kaomud Tyagi involved in visualization; Shantanu Kundu, Kaomud Tyagi, Vikas Kumar involved in writing—original draft; Shantanu Kundu, Vikas Kumar, Kailash Chandra involved in writing—review and editing.

## Data Availability

The following information was supplied regarding the availability of DNA sequences: The complete mitogenome of *Pangshura tentoria* is deposited in GenBank of NCBI under accession number MH795989.
