# Supplementary figures and images for: The first complete mitochondrial genome of the Indian Tent Turtle, Pangshura tentoria (Testudines: Geoemydidae): Characterization and comparative analysis
Source: Ecol Evol. 2019 Aug 30;9(18):10854–68. doi: 10.1002/ece3.5606 (PMC6787814; doi:10.1002/ece3.5606)

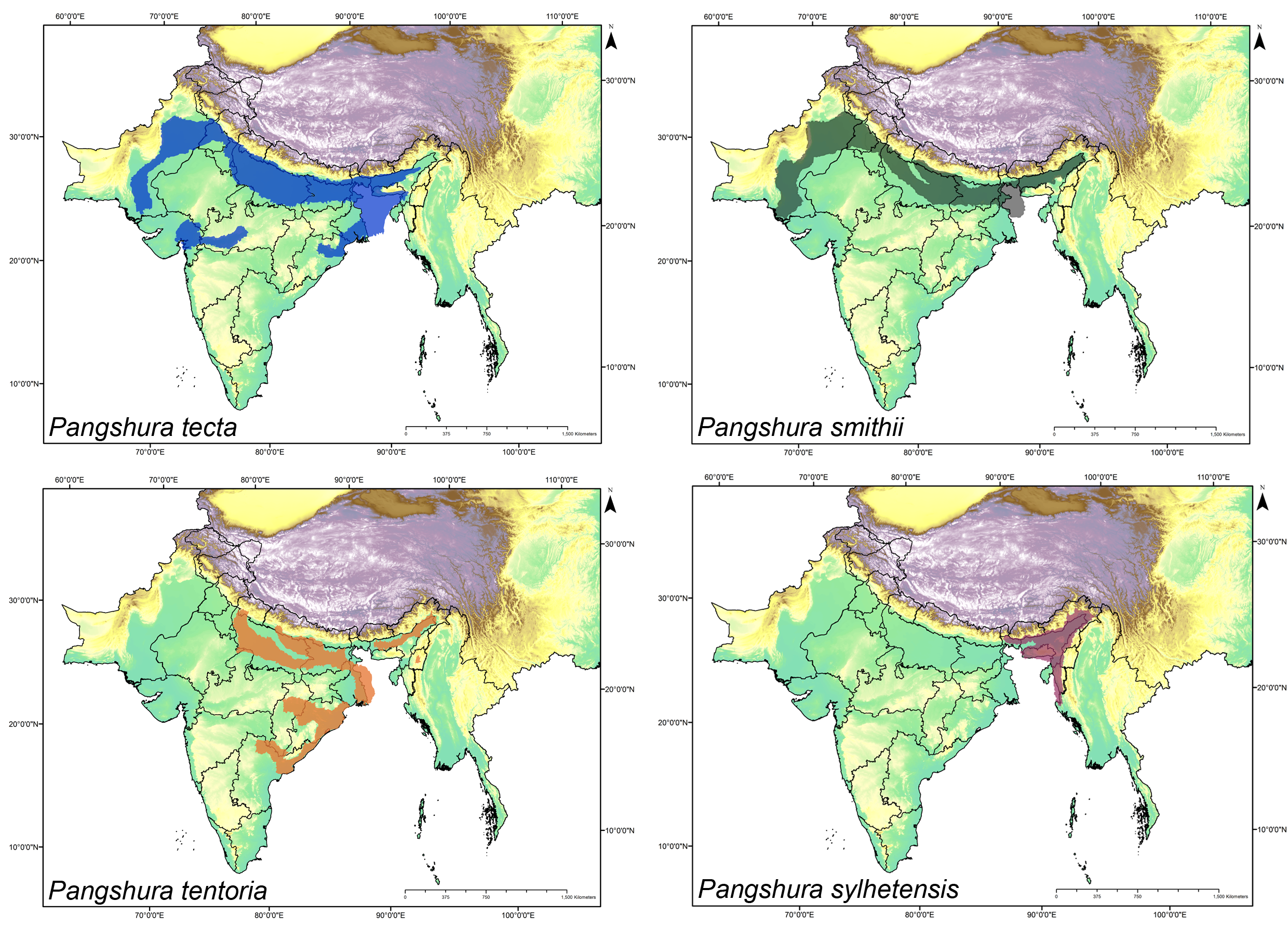

Supplement: Supplementary file 1 [file ECE3-9-10854-s001.tif]

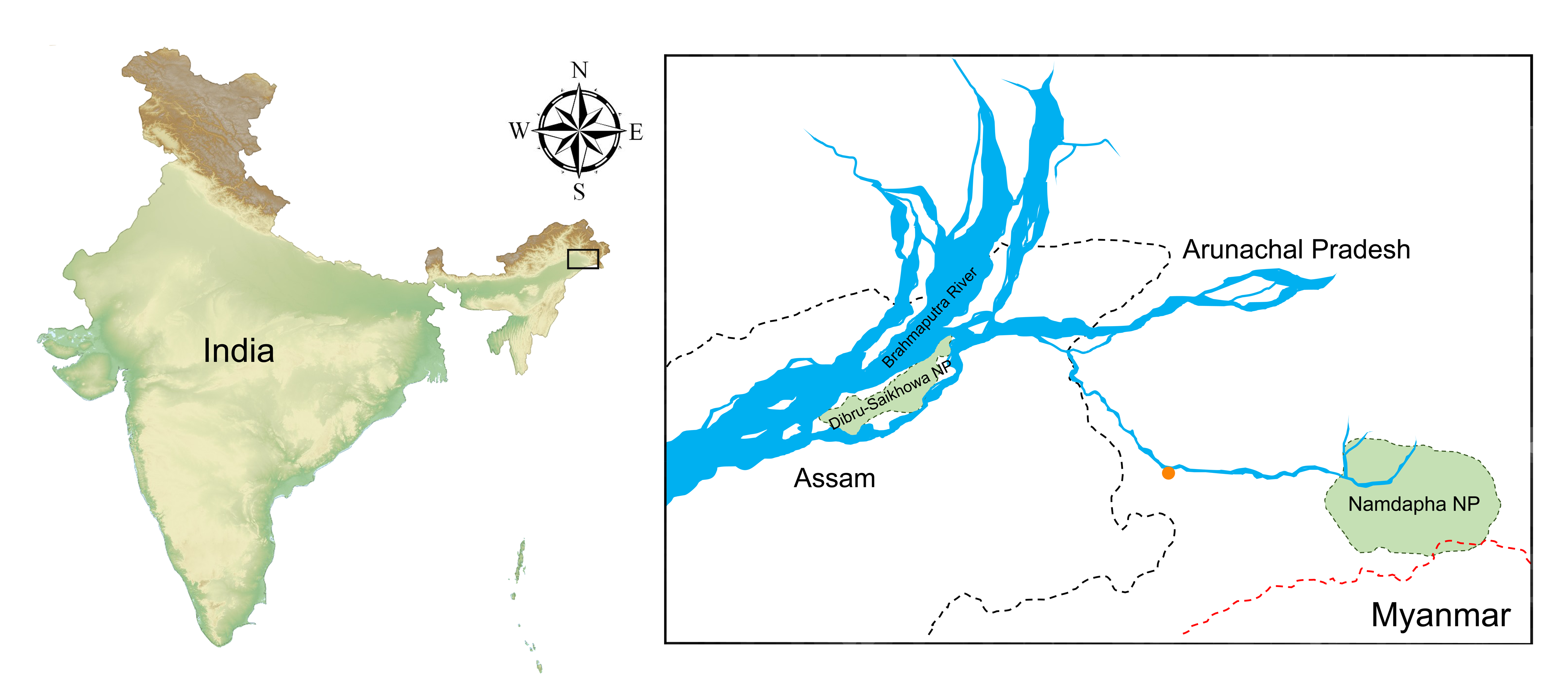

Supplement: Supplementary file 2 [file ECE3-9-10854-s002.tif]

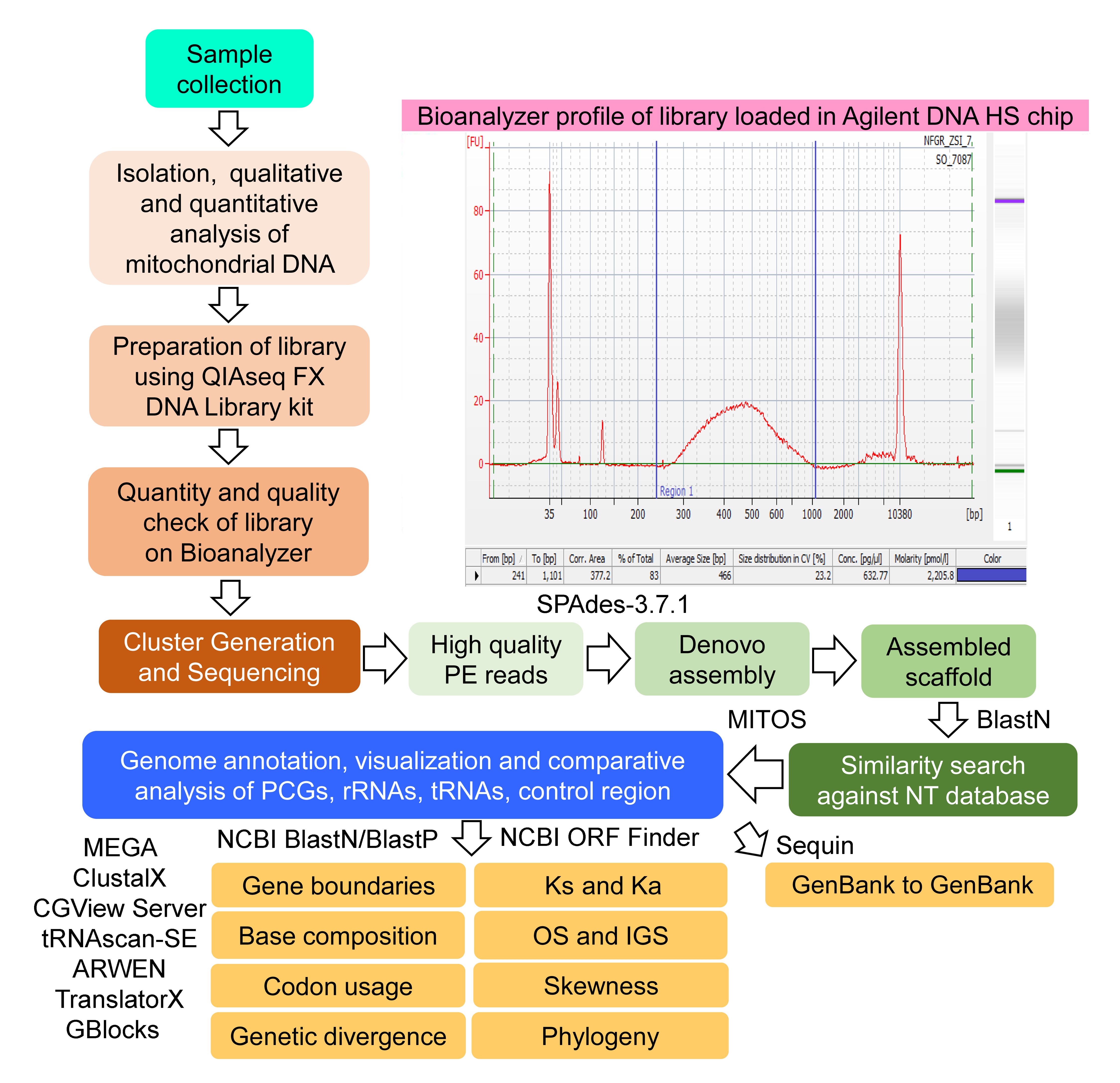

Supplement: Supplementary file 3 [file ECE3-9-10854-s003.tif]

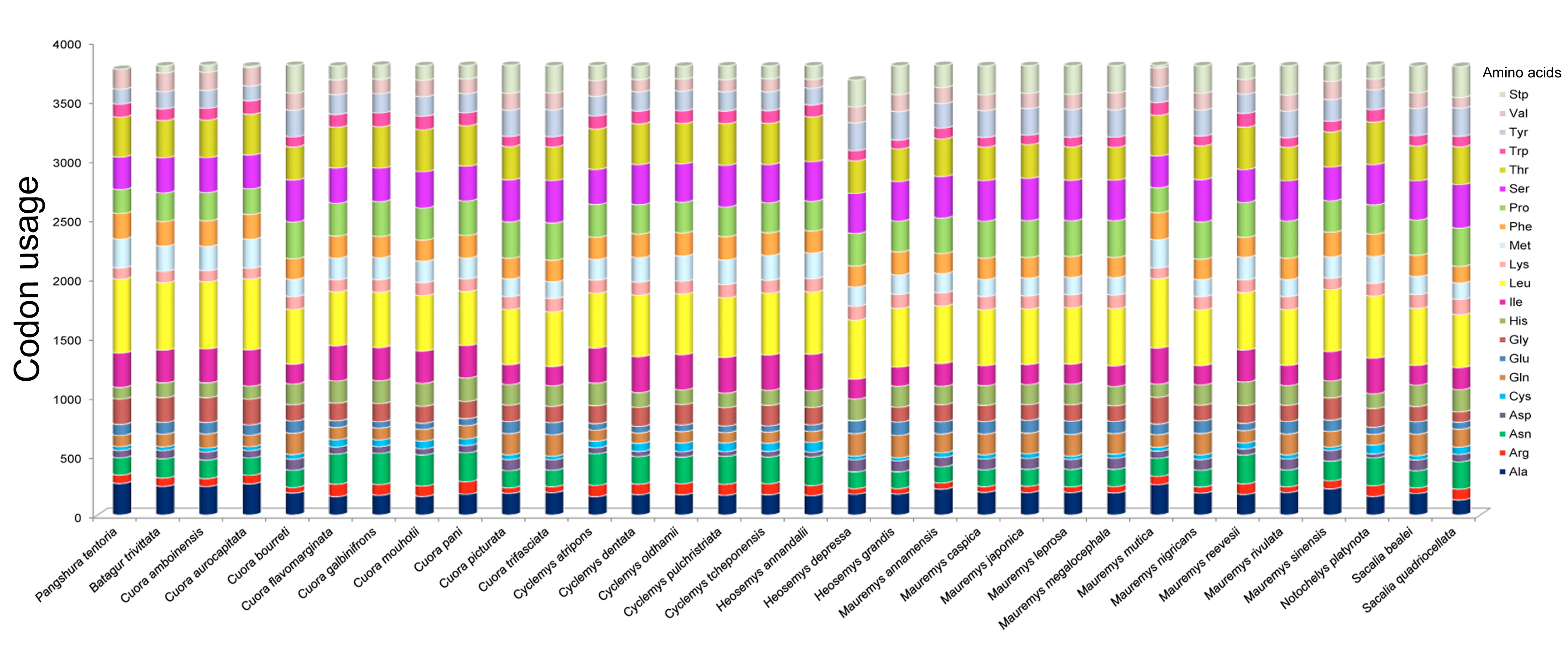

Supplement: Supplementary file 4 [file ECE3-9-10854-s004.tif]

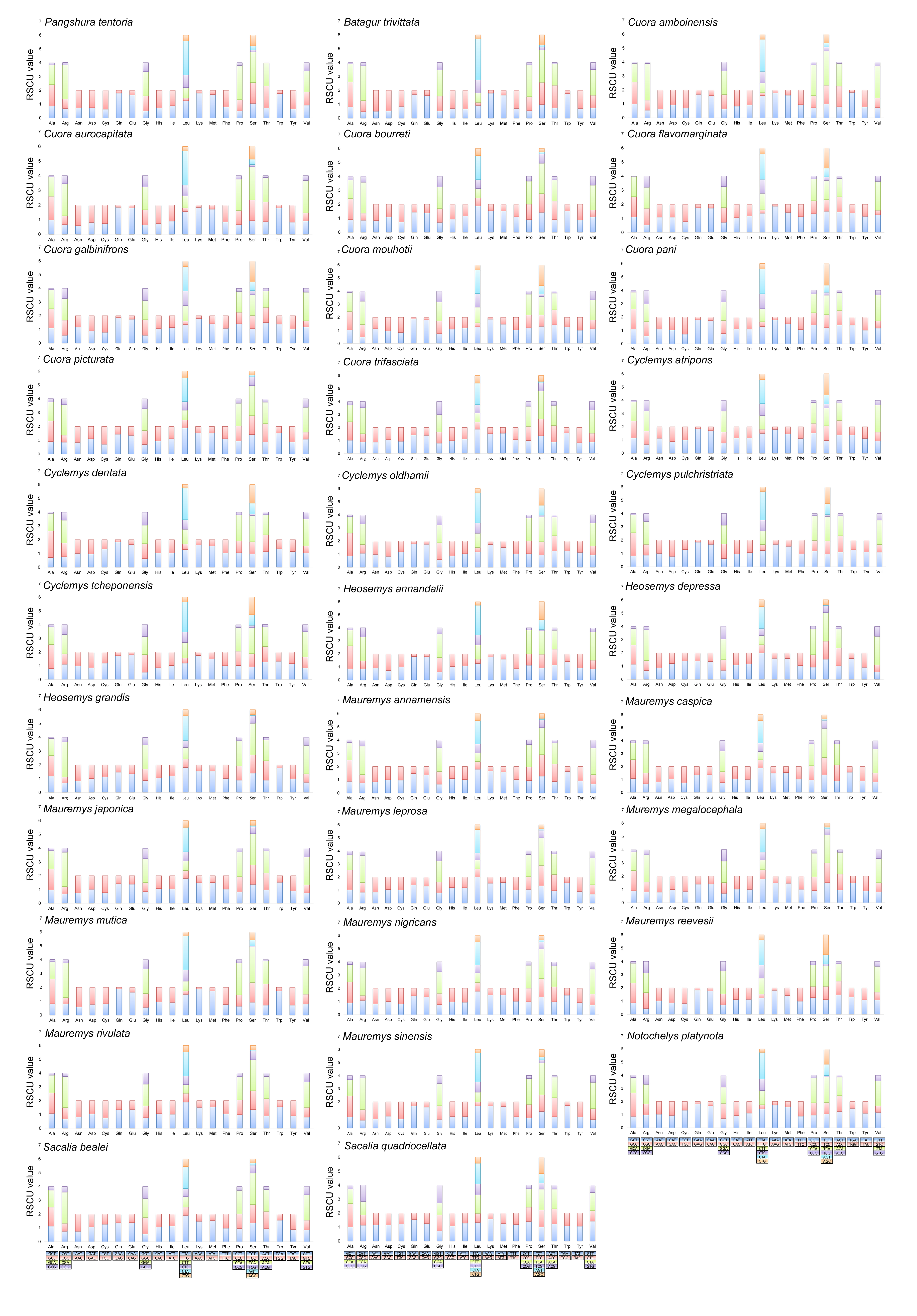

Supplement: Supplementary file 5 [file ECE3-9-10854-s005.tif]

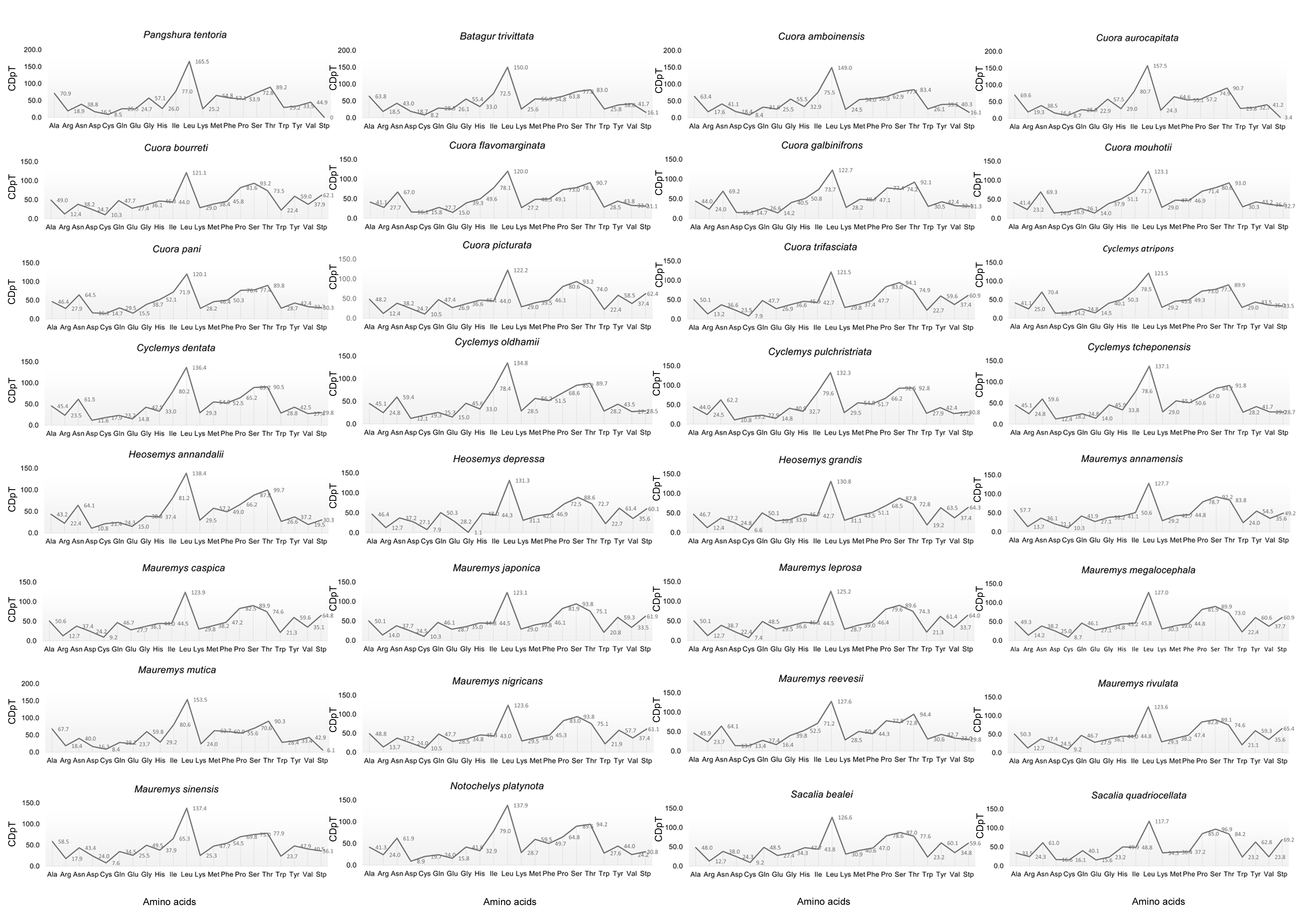

Supplement: Supplementary file 6 [file ECE3-9-10854-s006.tif]

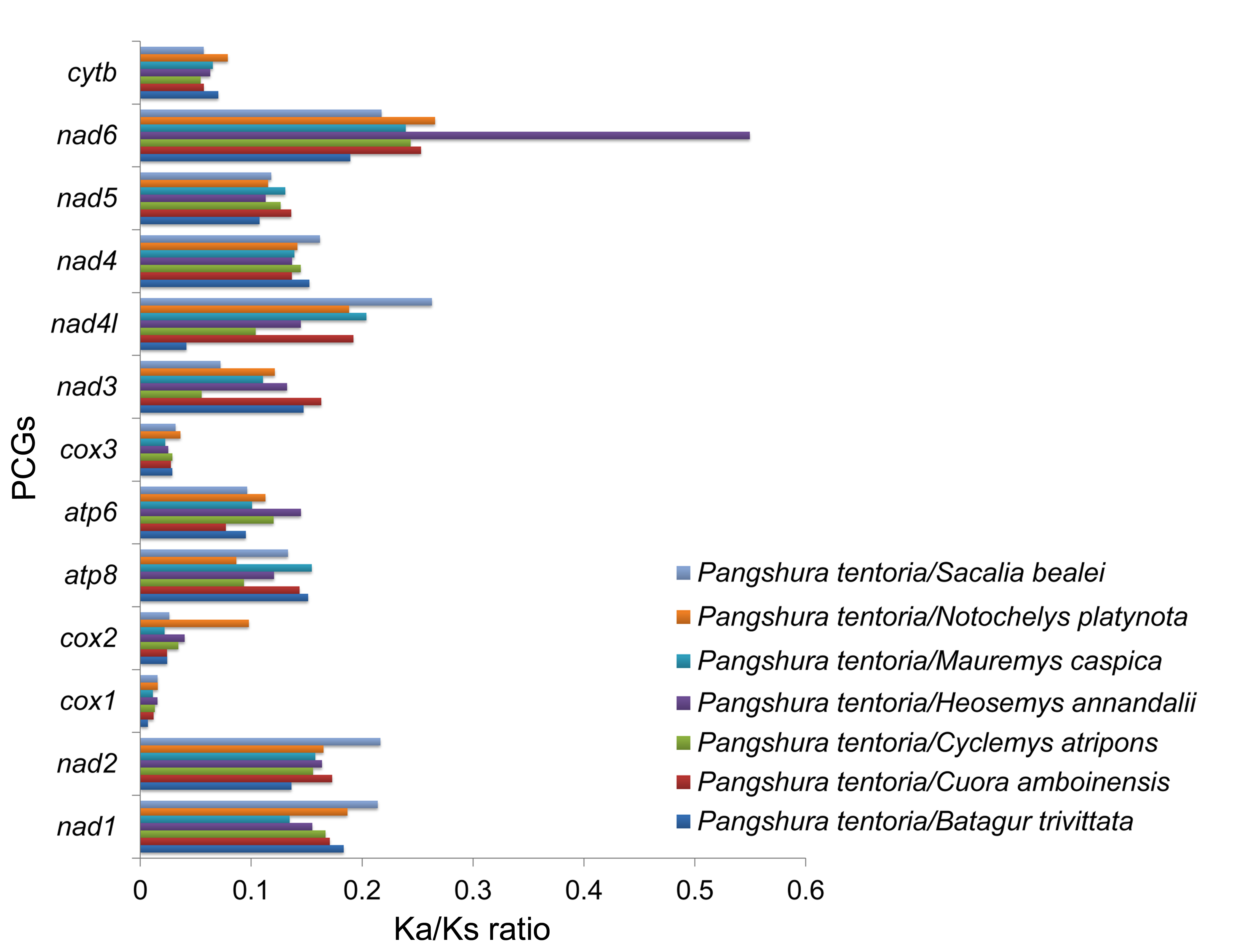

Supplement: Supplementary file 7 [file ECE3-9-10854-s007.tif]

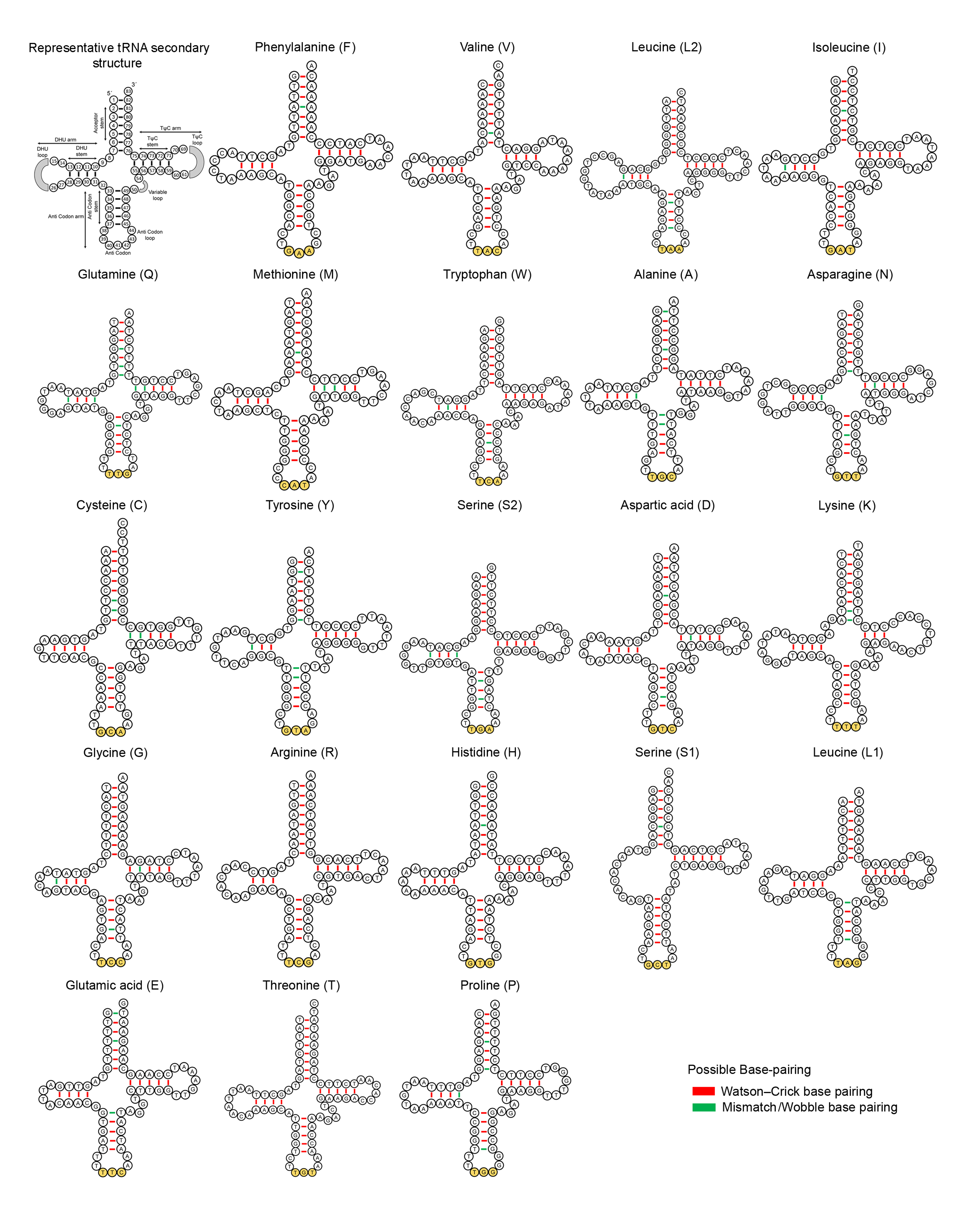

Supplement: Supplementary file 8 [file ECE3-9-10854-s008.tif]

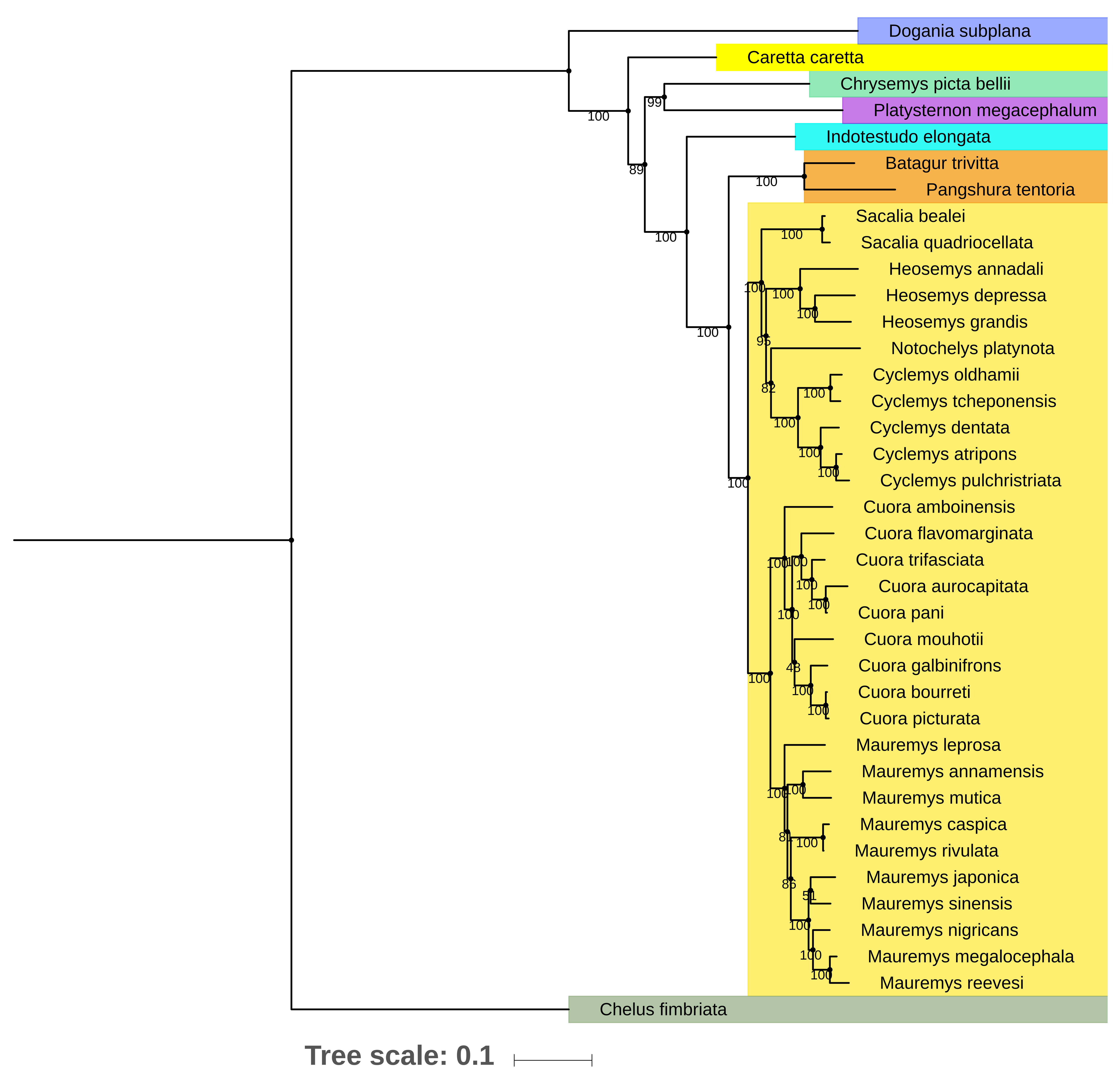

Supplement: Supplementary file 9 [file ECE3-9-10854-s009.tif]
