# Supplementary material for: The first complete mitochondrial genome of the Indian Tent Turtle, Pangshura tentoria (Testudines: Geoemydidae): Characterization and comparative analysis
Source: Ecol Evol. 2019 Aug 30;9(18):10854–68. doi: 10.1002/ece3.5606 (PMC6787814; doi:10.1002/ece3.5606)
Supplement: Supplementary file 10 [file ECE3-9-10854-s010.docx]

**Table S1. List of complete mitochondrial sequences of Geoemydidae species, acquired from the NCBI database used in the current comparative analysis.**

| **Sl. No.** | **Species Name** | **NCBI Accession No.** | **References** |
| --- | --- | --- | --- |
| 1 | *Pangshura tentoria* | MH795989 | This Study |
| 2 | *Batagur trivittata* | KX817298 | Feng et al. 2016 |
| 3 | *Cuora amboinensis* | FJ763736 | Unpublished |
| 4 | *Cuora aurocapitata* | AY874540 | Unpublished |
| 5 | *Cuora bourreti* | JN020145 | Unpublished |
| 6 | *Cuora flavomarginata* | EU708434 | Unpublished |
| 7 | *Cuora galbinifrons* | EU809939 | Unpublished |
| 8 | *Cuora mouhotii* | DQ659152 | Zhang et al. 2008 |
| 9 | *Cuora pani* | GQ889364 | Unpublished |
| 10 | *Cuora picturata* | JF712890 | Unpublished |
| 11 | *Cuora trifasciata* | KF574821 | Li et al. 2015 |
| 12 | *Cyclemys atripons* | EF067858 | Unpublished |
| 13 | *Cyclemys dentata* | JX455823 | Huang et al. 2015 |
| 14 | *Cyclemys oldhami* | JN582335 | Unpublished |
| 15 | *Cyclemys pulchristriata* | JQ266015 | Unpublished |
| 16 | *Cyclemys tcheponensis* | JQ277464 | Unpublished |
| 17 | *Heosemys annandalii* | JF742646 | Unpublished |
| 18 | *Heosemys depressa* | JQ266017 | Unpublished |
| 19 | *Heosemys grandis* | KX816868 | Unpublished |
| 20 | *Mauremys annamensis* | HM131942 | Unpublished |
| 21 | *Mauremys caspica* | KC692465 | Unpublished |
| 22 | *Mauremys japonica* | GU938833 | Unpublished |
| 23 | *Mauremys leprosa* | KP100055 | Unpublished |
| 24 | *Mauremys megalocephala* | HM132059 | Unpublished |
| 25 | *Mauremys mutica* | DQ453753 | Unpublished |
| 26 | *Mauremys nigricans* | KT951839 | Unpublished |
| 27 | *Mauremys reevesii* | AY676201 | Unpublished |
| 28 | *Mauremys rivulata* | KP100054 | Unpublished |
| 29 | *Mauremys sinensis* | FJ871126 | Unpublished |
| 30 | *Notochelys platynota* | HQ853256 | Unpublished |
| 31 | *Sacalia bealei* | GU183364 | Unpublished |
| 32 | *Sacalia quadriocellata* | EF088646 | Unpublished |
