# Supplementary material for: The first complete mitochondrial genome of the Indian Tent Turtle, Pangshura tentoria (Testudines: Geoemydidae): Characterization and comparative analysis
Source: Ecol Evol. 2019 Aug 30;9(18):10854–68. doi: 10.1002/ece3.5606 (PMC6787814; doi:10.1002/ece3.5606)
Supplement: Supplementary file 11 [file ECE3-9-10854-s011.docx]

**Table S2.** Estimated models by partitioning the 13 PCGs separately through PartitionFinder 2 for phylogenetic analysis.

| **Sl. No.** | **Model** | **PCGs** |
| --- | --- | --- |
| 1 | HKY+I+G | nad4l, atp8, nad4, atp6 |
| 2 | GTR+I+G | COII, COI, COIII |
| 3 | GTR+I+G | nad1, cytb |
| 4 | TIM+I+G | nad5, nad2 |
| 5 | HKY+G | nad3 |
| 6 | GTR+G | nad6 |
