# Supplementary material for: The first complete mitochondrial genome of the Indian Tent Turtle, Pangshura tentoria (Testudines: Geoemydidae): Characterization and comparative analysis
Source: Ecol Evol. 2019 Aug 30;9(18):10854–68. doi: 10.1002/ece3.5606 (PMC6787814; doi:10.1002/ece3.5606)
Supplement: Supplementary file 16 [file ECE3-9-10854-s016.docx]

**SUPPORTING INFORMATION**

**Table S1. List of complete mitochondrial sequences acquired from GenBank database used in the current comparative analysis.** (DOCX).

**Table S2. Estimated models by partitioning the 13 PCGs separately through PartitionFinder 2 for phylogenetic analysis.** (DOCX).

**Table S3. Comparision of Gene arrangements in light (-) and heavy (+) strand within 32 Geoemydidae species.** (XLS)

**Table S4. Comparison of overlapping and intergenic spacer regions of the studied 32 Geoemydid species.** The overlapping regions are denoted by minus digit. The gene arrangement of *B. trivittata* were mentioned in the right side. (XLS)

**Table S5. Start and stop codons of 13 PCGs in 32 Geoemydid species.** (XLS)

**Table S6. Anticodons of 22 tRNA genes in 32 Geoemydid species.** (XLS)

**Figure S1. The range distribution of four congeners of the genus *Pangshura*.** The country level topology map have been downloaded from DIVA-GIS Spatial data platform (<http://www.diva-gis.org/datadown>) and overlaying by ArcGIS 10.6 software(ESRI®, CA, USA). The distribution patterns were edited manually in Adobe Photoshop CS 8.0 followed by the published annotated checklist and atlas of taxonomy, synonymy, distribution, and conservation status of turtle of the world (TTWG, 2017).

**Figure S2. The sampling site of *P. tentoria* in northeast India.** Map not to scale and manually prepared by the first author (S.K.) with the help of Google Map (<https://www.google.com/maps>) and Adobe Photoshop CS 8.0. The riverine systems are marked by blue color, national boundary between state Arunachal Pradesh and Assam is marked by Black dotted line, international boundary of India and Myanmar is marked by Red dotted line, two National Parks are marked by Green colors, sampling site (27°30' N 95°59' E) is marked by orange dot.

**Figure S3. A pictorial overview of the methodologies used for sequencing and analysis of *P. tentoria* mitogenome and bioanalyzer profiles after sonication of enriched mitochondrial DNA sample and libraries.**

**Figure S4. Comparison of codon usage within the 32 Geoemydid species mitochondrial genome including *P. tentoria*.**

**Figure S5. Comparative Relative synonymous codon usage (RSCU) in 32 Geoemydid species including *P. tentoria*.** The cumulative RSCU values are represented on the y-axis while the codon families for each amino acid are represented on the x-axis.

**Figure S6. Codon distribution 32 Geoemydid species including *P. tentoria*. CDspT=codons per thousand codons.**

**Figure S7. Ka/Ks ratios for the 13 mitochondrial protein-coding genes among *P. tentoria* and other Geoemydid species representing seven genera.**

**Figure S8. Putative secondary structures for 22 tRNA genes in mitochondrial genome of *P. tentoria*.** The first structure shows the nucleotide positions and details of stem-loop of tRNAs. The tRNAs are represented by full names and IUPAC-IUB single letter amino acid codes. Watson-Crick, wobble, and mismatch base pairing are shown by red and green color bars respectively. The secondary structure of tRNAs were predicted by ARWEN 1.2 online server and edited manually in Adobe Photoshop CS 8.0.

**Figure S9. Maximum Likelihood (ML) phylogenetic tree of 32 Geoemydid species showing the evolutionary relationship of *P. tentoria*.** The ML tree was drawn by IQ-Tree in CIPRES Science Gateway V. 3.3 and edited with iTOL v4 and Adobe Photoshop CS 8.0.
